# Neuroprotective Role of Dietary Supplementation with Omega-3 Fatty Acids in the Presence of Basal Forebrain Cholinergic Neurons Degeneration in Aged Mice

**DOI:** 10.3390/ijms21051741

**Published:** 2020-03-04

**Authors:** Debora Cutuli, Eugenia Landolfo, Davide Decandia, Annalisa Nobili, Maria Teresa Viscomi, Livia La Barbera, Stefano Sacchetti, Paola De Bartolo, Annacarmen Curci, Marcello D’Amelio, Stefano Farioli-Vecchioli, Laura Petrosini

**Affiliations:** 1 IRCCS Fondazione Santa Lucia, 00143 Rome, Italy; eugenia.landolfo@uniroma1.it (E.L.); davide.decandia91@gmail.com (D.D.); a.nobili@hsantalucia.it (A.N.); mt.viscomi@hsantalucia.it (M.T.V.); livia.labarbera@gmail.com (L.L.B.); p.debartolo@unimarconi.it (P.D.B.); m.damelio@hsantalucia.it (M.D.); laura.petrosini@uniroma1.it (L.P.); 2 Department of Psychology, University of Rome “Sapienza”, 00185 Rome, Italy; stefano.sacchetti@uniroma1.it (S.S.); annacarmencurci@gmail.com (A.C.); 3 Department of Medical and Surgical Sciences, University “Campus Biomedico”, 00128 Rome, Italy; 4 Department of Life Science and Public Health section of Histology and Embryology, Università Cattolica del Sacro Cuore, 00168 Rome, Italy; 5 Department of Human Sciences, Guglielmo Marconi University, 00193 Rome, Italy; 6 Institute of Biochemistry and Cell Biology, CNR, 00015 Monterotondo, Italy; stefano.fariolivecchioli@cnr.it

**Keywords:** aging, cholinergic system, omega-3 fatty acids, prevention, cognitive deficits, neuroprotection

## Abstract

As major components of neuronal membranes, omega-3 polyunsaturated fatty acids (n-3 PUFA) exhibit a wide range of regulatory functions. Recent human and animal studies indicate that n-3 PUFA may exert beneficial effects on aging processes. Here we analyzed the neuroprotective influence of n-3 PUFA supplementation on behavioral deficits, hippocampal neurogenesis, volume loss, and astrogliosis in aged mice that underwent a selective depletion of basal forebrain cholinergic neurons. Such a lesion represents a valid model to mimic a key component of the cognitive deficits associated with dementia. Aged mice were supplemented with n-3 PUFA or olive oil (as isocaloric control) for 8 weeks and then cholinergically depleted with mu-p75-saporin immunotoxin. Two weeks after lesioning, mice were behaviorally tested to assess anxious, motivational, social, mnesic, and depressive-like behaviors. Subsequently, morphological and biochemical analyses were performed. In lesioned aged mice the n-3 PUFA pre-treatment preserved explorative skills and associative retention memory, enhanced neurogenesis in the dentate gyrus, and reduced volume and VAChT levels loss as well as astrogliosis in hippocampus. The present findings demonstrating that n-3 PUFA supplementation before cholinergic depletion can counteract behavioral deficits and hippocampal neurodegeneration in aged mice advance a low-cost, non-invasive preventive tool to enhance life quality during aging.

## 1. Introduction

Given dementia is a major cause of death and disability in older population and no effective pharmacological treatment has been identified to date, there is considerable interest in identifying lifestyle approaches, such as diet, able to prevent cognitive decline during aging [1,2,3,4]. Among the different forms of dementia, Alzheimer’s disease (AD) is the most common (60–70% of cases) and currently affects 47 million people worldwide [5,6,7]. Its prevalence rises exponentially with age and, due to increasing lifespan, it has been predicted to double every 20 years, causing a huge burden on healthcare costs [5,8]. AD is characterized by irreversible and progressive brain atrophy, loss of memory, and cognition. Specifically, basal forebrain cholinergic neurons degeneration and the subsequent loss of cholinergic neurotransmission in the cerebral cortex and limbic system are retained pathophysiological events crucial in triggering the cognitive deterioration observed in patients with AD dementia [9,10].

In the past three decades, the availability of saporin immunotoxins allowed studying the role of basal forebrain cholinergic system in several cognitive functions and its implications in aging and dementia [11,12,13]. In fact, saporin immunotoxins selectively cause death of cholinergic cells by inhibiting ribosomal protein synthesis when it is taken up into cells expressing the low-affinity p75 neurotrophin receptor [11,14,15]. The resulting permanent and selective saporin-dependent massive loss of cholinergic basal forebrain neurons mimics neuropathological features and cognitive symptoms associated with mild cognitive impairment (MCI) and mild AD.

In the present study we used the mu-p75-saporin (sap) immunotoxin intracerebroventricularly injected in aged mice to elicit the basal forebrain cholinergic depletion. In the experimental model of first stages of AD so obtained we analyzed the neuroprotective properties of pre-lesional treatment with omega-3 polyunsaturated fatty acids (n-3 PUFA).

n-3 PUFA are one the major components of neuronal membranes and key modulators of neuroinflammation, oxidative stress, and neurogenesis [16,17]. They include eicosapentaenoic acid (EPA), docosahexaenoic acid (DHA), docosapentaenoic acid (DPA) and alpha-linolenic acid (ALA) [16,18]. Longer-chain n-3 fatty acids (EPA and DHA) are synthetized by shorter-chain n-3 fatty acids (ALA) [19,20]. However, biological conversion is inefficient, especially during aging [19,21,22,23,24]. In addition, shorter-chain fatty acids cannot be synthesized by humans [21,25]. Therefore, diet is the most important source of these fatty acids. Their daily intake could be from plant-derived ALA and from fish and marine EPA and DHA, and their supplements [26]. Unfortunately, nutritional research indicates that the “Western pattern diet” does not provide the aged brain with an optimal supply of n-3 PUFA, and aging *per se* is associated with a decrease in cerebral n-3 PUFA [27].

Notably, n-3 PUFA are reported to exert beneficial and neuroprotective effects on the aging brain [23], when deterioration in neuronal function and decline in cognitive performance, mainly those hippocampal-dependent, have been consistently reported. These age-related impairments are reflective of synaptic loss, decreased neurogenesis, synaptic plasticity, neuronal density, and gray matter volume, particularly in the hippocampal circuits [28,29,30,31,32,33]. Other studies indicate that deficits in hippocampal functions are associated with neuroinflammation and oxidative stress [33,34,35,36]. Interestingly, experimental studies in rodents have shown on one hand that n-3 PUFA supplementation improves neurogenesis and synaptogenesis, as well as executive functions and learning abilities, and on the other hand that n-3 PUFA deficiency is associated with memory deficits and impaired hippocampal plasticity [2,3,16,37,38]. Preclinical evidence from our laboratory confirmed that age-related alterations may lead to irreversible neuronal loss of gray matter volume in the hippocampus and prefrontal lobes [39,40], in line with previous studies in humans [41,42,43]. Specifically, we demonstrated that 8-week n-3 PUFA supplementation in aged mice robustly ameliorates mnesic functions and coping skills via increased neurogenesis and reduced hippocampal neurodegenerative processes [39], in association with foci of greater gray matter volume in fronto-hippocampal areas [39,40].

Human longitudinal studies based on direct or indirect indices of n-3 PUFA consumption correlate with better cognitive functioning and reduced risk of dementia, higher total brain and regional gray matter volumes [44,45,46,47,48,49] and reduced white matter hyperintensity [50,51]. Some interventional studies reported that n-3 PUFA supplementation improves cognition in healthy elderly subjects [52,53,54] and in subjects with MCI [55,56,57,58]. Many reports have also demonstrated the benefits of a diet rich in n-3 PUFA, as the Mediterranean diet, against age-related cognitive decline in MCI subjects and AD patients [59,60,61,62,63,64,65]. Anyway, still little is known about the brain mechanisms and correlates of the preserved cognitive functions in relation to the preventive effects of n-3 PUFA dietary intake during aging.

**Figure 1:**
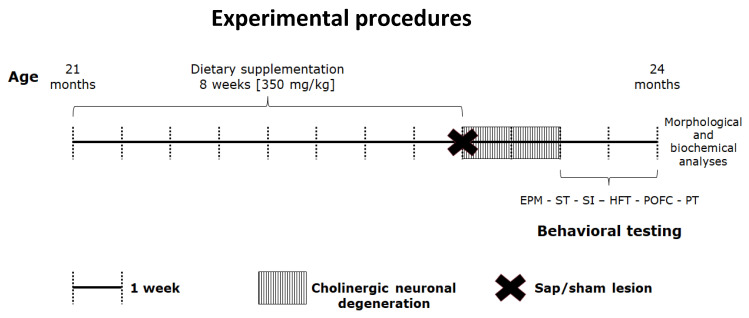
Experimental procedures. After 8-week oral supplementation with n-3 PUFA, 21-month old aged mice have been subjected to intracerebroventricular (i.c.v.) injections of mu-p75-saporin or saline (sham lesion) to selectively deplete the forebrain cholinergic system. Two weeks after the lesion, the animals were behaviorally tested by means of validated tasks (Elevated Plus Maze, EPM; Splash Test, ST; Social Interactions, SI; Hidden Food Test, HFT; Predator Odor Fear Conditioning, POFC; Porsolt Test, PT). At the end of testing battery, mice were sacrificed, and brains collected for morphological and biochemical analyses.

To this end, here we focused on the neuroprotective action of n-3 PUFA by investigating the influence of an 8-week oral pre-lesional treatment with a mixture of EPA, DHA, and DPA on the behavioral deficits and hippocampal degeneration induced by immunotoxic forebrain cholinergic lesions during aging. To this aim, emotional, motivational, social and mnesic performance as well as hippocampal morphological and biochemical correlates of cholinergically depleted aged mice pre-treated with n-3 PUFA or olive oil (used as isocaloric control) were compared with those of pre-treated with n-3 PUFA or olive oil sham-lesioned animals (Figure 1). After behavioral testing, neurodegeneration of hippocampal networks was analyzed by measuring neurogenesis levels in the dentate gyrus (DG) as well as volumes and astrogliosis in the hippocampus, which is one of the main projection areas of the lesioned cholinergic projections from medial septum/diagonal band.

## 2. Results

### 2.1. Behavioral Testing

#### 2.1.1. Elevated Plus Maze (EPM)

Since anxiety is reported to increase in aging rodents [66] and cholinergic manipulations are known to influence anxiety levels [67,68], in the present study we used the EPM as a validated test to measure anxiety in rodents based on their natural aversion for heights and open spaces [39,40,69,70].

After a square root transformation (to adjust for normality), duration and frequency EPM data were analyzed by three-way analyses of variance (ANOVA) (diet x lesion x arm). Defecations were not normally distributed.

As for the duration of time spent in the closed vs. open arms, the ANOVA revealed a significant arm effect (F_1,38_ = 51.56, *p* < 0.000001), while diet (F_1,38_ = 0.06, *p* = 0.81) and lesion (F_1,38_ = 1.11, *p* = 0.30) effects were not significant. The interactions diet x lesion (F_1,38_ = 0.13, *p* = 0.72), arm x diet (F_1,38_ = 0.12, *p* = 0.74), arm x lesion (F_1,38_ = 0.39, *p* = 0.54), arm x diet x lesion (F_1,38_ = 0.22, *p* = 0.64) were not significant.

**Figure 2:**
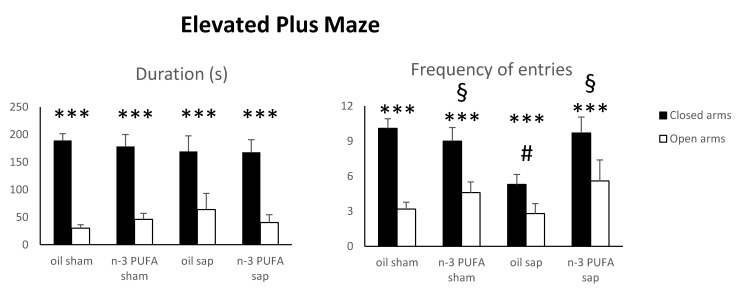
Elevated Plus Maze data. Duration and frequency of exploration of the closed and open arms in the four experimental groups. Data are reported as mean and SEM (oil sham, n = 12; n-3 PUFA sham, n = 10; oil sap, n = 10; n-3 PUFA sap, n = 10). Arm effect: *** *p* < 0.000001; Diet effect: § *p* < 0.05; Diet x lesion effect: # *p* < 0.05.

The ANOVA performed on frequency of entries in the closed vs. open arms showed significant diet (F_1,38_ = 5.76, *p* = 0.02) and arm (F_1,38_ = 40.96, *p* < 0.000001) effects as well as a significant first level diet x lesion interaction (F_1,38_ = 4.29, *p* = 0.04). Lesion effect (F_1,38_ = 1.81, *p* = 0.19) and interactions arm x diet (F_1,38_ = 0.68, *p* = 0.41), arm x lesion (F_1,38_ = 1.31, *p* = 0.26) arm x diet x lesion (F_1,38_ = 0.53, *p* = 0.47) were not significant. Post-hoc comparisons calculated on the significant interaction demonstrated that the oil sap group explored the arms less frequently than the remaining groups (oil sap vs. oil sham, *p* = 0.02; oil sap vs. n-3 PUFA sham, *p* = 0.03; oil sap vs. n-3 PUFA sap, *p* = 0.02; Figure 2).

No differences were found in the number of defecations (Kruskal–Wallis test; H = 3.08, *p* = 0.38).

These findings indicate that n-3 PUFA pre-treatment was able to increase explorative behavior (frequency of entries) and to prevent its lesion-induced reduction, while the expected preference for the closed arms was kept unaltered by both dietary and lesional treatments.

#### 2.1.2. Splash Test (ST)

The ST is based on the assessment of grooming behavior considered to be a form of self-care/motivational behavior that parallels with some symptoms of depression, such as apathetic behavior [71], and it is associated with hedonic reactivity in the sucrose preference test and increased immobility in the forced swim test [72,73].

No differences were found in self-care and motivational behaviors among groups (Supplementary Materials Figure S1). In particular, as demonstrated by Kruskal–Wallis analysis, mice belonging to all experimental groups showed not significantly different duration (H = 2.39, *p* = 0.49) and frequency (H = 5.52, *p* = 0.14) of grooming. The number of defecations was also similar in all groups (H = 2.35, *p* = 0.50).

#### 2.1.3. Social Interactions (SI)

The SI test is used to investigate social behaviors, which are known to decrease with age in rodents [66,74].

Since most behaviors displayed in SI test were sporadic and not normally distributed, we analyzed social interaction data by means of non-parametric analysis. Sexual behaviors were not observed in any group of mice.

No differences were found among groups in total duration (H = 1.67, *p* = 0.64) and frequency (H = 3.15, *p* = 0.37) of social behaviors, as well as in total duration (H = 0.89, *p* = 0.83) and frequency (H = 6.92, *p* = 0.07) of non-social behaviors (Supplementary Materials Figure S2A,B). No significant differences were observed when single social or non-social behaviors were analyzed (Supplementary Materials Table S1). No differences were evident in the number of defecations of the four experimental groups (H = 0.68, *p* = 0.88).

#### 2.1.4. Hidden Food Test (HFT)

The HFT checks whether food-deprived mice can find the pleasant food pellet hidden beneath the cage’s bedding to uncover eventual deficits in olfactory abilities [75]. Impairments in the sense of smell are common during aging and may be due to the deterioration of the peripheral sensory epithelium or central olfactory relays as well as to degenerative processes affecting cognitive processing of odors in the brain [76]. Age-related neurodegenerative diseases, such as AD, seem to involve selective pathology in specific brain structures linked to olfactory processing [77]. In addition, in the present study we verified the olfactory capabilities within experimental mice groups because the sensitivity to odors was relevant to perform the subsequent POFC test.

No differences between latency to dig out and latency to eat the palatable food pellet during the HFT were found in all experimental groups (Mann-Whitney U test; U = 0, *p* = 1), thus we collapsed the two parameters (linked to sensory odor perception and motivation, respectively) by analyzing the average time to dig out and eat the palatable food among groups. As demonstrated by Kruskal–Wallis analysis, no differences in mean latency to dig out and eat the palatable food pellet (H = 2.97, *p* = 0.39) were found among the four experimental groups (Supplementary Materials Figure S3).

These findings indicate both the preserved ability to smell volatile odors and the comparable motivation levels in lesioned and sham animals regardless of their pre-treatment.

#### 2.1.5. Predator Odor Fear Conditioning (POFC)

The POFC involves the use of the predator odor as a natural unconditioned fear stimulus (instead of an aversive electric foot-shock) to assess the integrity of hippocampal networks associated with associative learning and memory [78,79]. It has been previously reported that in mice the exposure to predator odor (e.g., coyote urine) is able to activate place cells in CA1 and modify their firing patterns to establish a spatial representation of the fearful experience [80]. Also, research on AD patients showing early atrophy of medial temporal lobe structures indicated marked impairments in fear conditioning [81,82].

Since one oil sham mouse died before testing, the number of animals belonging to oil sham group performing this test was 11. As expressed by percentages, the retention index was analyzed by a two-way ANOVA (diet x lesion) after angular transformation {arcsine [square root(percentage of freezing/100)]}. Freezing time during exposure session was analyzed by non-parametric analyses due to the lack of normality distribution of the data.

**Figure 3:**
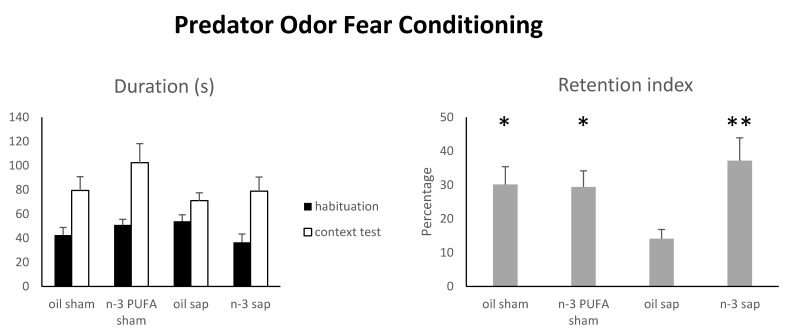
Predator Odor Fear Conditioning. Duration of freezing in the four experimental groups during habituation and context test sessions were used to calculate the retention index. Data are reported as mean and SEM (oil sham, n = 11; n-3 PUFA sham, n = 10; oil sap, n = 10; n-3 PUFA sap, n = 10). Asterisks indicate the level of statistical significance of the post-hoc comparisons between oil sap and the remaining groups: * *p* < 0.05, ** *p* < 0.01.

The two-way ANOVA (diet x lesion) revealed a significant diet x lesion interaction (F_1,37_ = 4.84, *p* = 0.03), while diet (F_1,37_ = 4.06, *p* = 0.05) and lesion (F_1,37_ = 0.76, *p* = 0.39) effects were not significant. Post-hoc comparisons demonstrated that n-3 PUFA pre-treatment ameliorated the retention of aversive contextual memory in cholinergically depleted aged mice (Figure 3). In fact, lesioned aged mice pre-treated with omega-3 (n-3 PUFA sap) displayed similar retention index values in comparison to both sham groups (n-3 PUFA sap vs. oil sham, *p* = 0.42; n-3 PUFA sap vs. n-3 PUFA sham, *p* = 0.62). On the contrary, lesioned aged mice pre-treated with olive oil (oil sap) showed inferior retention index values either when compared to lesioned aged mice pre-treated with n-3 PUFA (*p* = 0.008) and to both sham control groups (oil sham, n-3 PUFA sham, *p* = 0.04). The retention indices of the two sham-lesioned aged groups (oil sham and n-3 PUFA sham) did not differ (*p* = 0.90).

No differences were evident in freezing time during exposure session (Kruskal–Wallis test; H = 3.68, *p* = 0.30; Supplementary Materials Figure S4) and in the number of defecations (habituation: H = 0.97, *p* = 0.81; exposure: H = 3.03, *p* = 0.39; context test: H = 1.86, *p* = 0.60) among the four experimental groups.

#### 2.1.6. Porsolt Test (PT)

The PT is a validated test measuring coping strategies and depressive-like behaviors when the animals are faced with an acute inescapable aversive situation [83,84,85,86]. Currently there is a growing interest in the relation between coping and depression in older people, but research on this issue is still scarce and systematic reviews are lacking [87,88,89].

Following a square root transformation (to adjust for normality), Porsolt Test data were analyzed by three-way ANOVAs (diet x lesion x strategy).

As for the duration of active vs. passive coping strategies a three-way ANOVA (diet x lesion x strategy) revealed a significant strategy effect (F_1,37_ = 205.32, *p* < 0.000001), while the remaining main factors and interactions were not significant (diet: F_1,37_ = 0.81, *p* = 0.37; lesion: F_1,37_ = 0.77, *p* = 0.39; diet x lesion: F_1,37_ = 0.12, *p* = 0.73; strategy x diet: F_1,37_ = 0.40, *p* = 0.53; strategy x lesion: F_1,37_ = 2.80, *p* = 0.10; strategy x diet x lesion: F_1,37_ = 0.01, *p* = 0.92).

A three-way ANOVA (diet x lesion x strategy) on frequency of active vs. passive coping strategies revealed a significant strategy effect (F_1,37_ = 27.38, *p* = 0.000007) while the remaining main factors and interactions were not significant (diet: F_1,37_ = 0.42, *p* = 0.52; lesion: F_1,37_ = 0.08, *p* = 0.77; diet x lesion: F_1,37_ = 0.02, *p* = 0.90; strategy x diet: F_1,37_ = 0.08, *p* = 0.77; strategy x lesion: F_1,37_ = 0.02, *p* = 0.88; strategy x diet x lesion: F_1,37_ = 0.04, *p* = 0.83).

**Figure 4:**
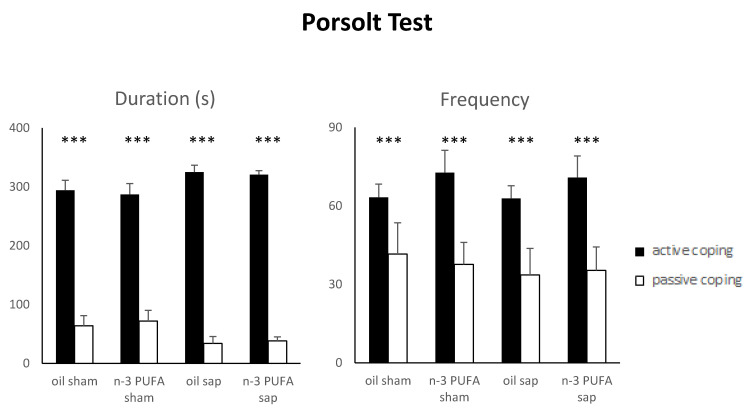
Porsolt Test. Duration and frequency of active and passive coping strategies displayed by the four experimental groups. Data are reported as mean and SEM (oil sham, n = 11; n-3 PUFA sham, n = 10; oil sap, n = 10; n-3 PUFA sap, n = 10). Strategy effect: *** *p* < 0.00001.

Such findings demonstrate that all mice (regardless of dietary or lesional treatment) faced the acute stress condition by adopting active coping strategies (Figure 4). The single strategies did not differ in duration and frequency among groups (Supplementary Materials Table S2).

### 2.2. Morphological Analyses

**Figure 5:**
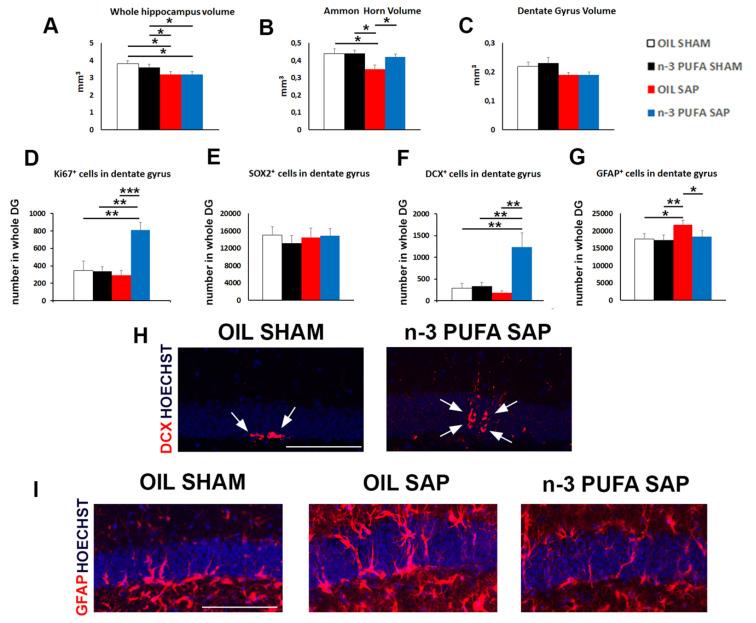
Morphological results. (**A**–**C**). Diagrams showing the variations of volume in the whole hippocampus, Ammon’s Horn (CA1 + CA3) and Dentate Gyrus (DG) in the four experimental groups (n = 5 mice/group). (**D**). Graph showing the large increase of Ki67-positive neuroblasts in the n-3 PUFA sap group in respect to the other groups. (**E**). Graph representing the unchanged values detected in the SOX2^+^ sub-populations. (**F**). Diagram showing the enhancement of DCX-positive neuroblasts in the n-3 PUFA sap group in respect to the other groups. (**G**). Histogram indicating the GFAP^+^ cell number in the DG. (**H**). Representative images showing the increase in the DCX^+^ cells (red, arrows) in the DG of the n-3 PUFA sap mice, when compared with oil sham group. (**I**). Representative fluorescence images of GFAP^+^ cells (red), showing the increased astrogliosis in the oil sap group, counteracted by the pre-treatment with n-3 PUFA. Scale bar 100 µm. * *p* < 0.05, ** *p* < 0.01, *** *p* < 0.001; Student’s *t* test.

Lesioned aged mice pre-treated with olive oil (oil sap) showed a significant decrease of the total hippocampal volume in comparison to the sham groups pre-treated with oil and n-3 PUFA (oil sap vs. oil sham and vs. n-3 PUFA sham *p* = 0.04, Figure 5A). The treatment with n-3 PUFA was not able to counteract the sap-dependent reduction of the total hippocampal volume (n-3 PUFA sap vs. oil sham and vs. n-3 PUFA sham *p* = 0.04, Figure 5A). A deeper analysis of the volume of the different hippocampal sub-regions, the Ammon’s Horn (CA1 + CA3) and DG, demonstrated that n-3 PUFA pre-treatment counteracted the decrease in Ammon’s Horn volume observed in the oil sap group in comparison to the both sham groups (oil sap vs. oil sham and vs. n-3 PUFA sham *p* = 0.01, n-3 PUFA sap vs. oil sap *p* = 0.02, Figure 5B). Conversely, in the DG we did not observe any significant difference among groups (Figure 5C).

### 2.3. Hippocampal Neurogenesis and Astrogliosis

#### 2.3.1. Neural Stem Cells and Proliferation

In DG of the adult hippocampus, new neurons originate from adult Neural Stem Cells (NSCs) by through a complex mechanism comprising different steps of proliferation and differentiation of NSCs and neural progenitors. However, in aged mice a severe decrease of progenitor proliferation has been observed, resulting in progressive decline of adult neurogenesis. To evaluate the effect of n-3 PUFA pre-treatment on the hippocampal neurogenesis of immunotoxically or sham-lesioned mice, we investigated the proliferation (detected by the proliferation marker Ki67) and differentiation (detected by the SOX2 and doublecortin (DCX) markers) of newborn neurons in the four experimental groups. In the n-3 PUFA sap group we observed a striking increase of cell proliferation respect to the other experimental groups (n-3 PUFA sap vs. oil sham, *p* = 0.005, vs. n-3 PUFA sham *p* = 0.001, vs. oil sap *p* = 0.0001, Figure 5D). To evaluate which sub-population of NSCs/neural progenitors might contribute to the increased proliferation observed in the n-3 PUFA sap, we used: SOX2, which specifically labels the NSCs and the early differentiating progenitors, and doublecortin (DCX), which is expressed by the late differentiating and post-mitotic neuroblast. While the analysis of SOX2 expression did not reveal any significant variation of the NSCs/neural progenitors among groups (Figure 5E), a large enhancement of the DCX^+^ sub-population in the n-3 PUFA sap mice was observed in comparison to the other experimental groups (n-3 PUFA sap vs. oil sham and vs. n-3 PUFA sham *p* = 0.01, vs. oil sap *p* = 0.006, Figure 5F,H).

#### 2.3.2. Hippocampal Astrogliosis

A specific hallmark of injury-dependent neuroinflammation is represented by increased GFAP^+^ astrocytic population, a process named astrogliosis. Our data showed that sap lesion induced a striking increase of GFAP^+^ astrocytes in the oil pre-treated aged mice (oil sap vs. oil sham *p* = 0.01). However, the administration of n-3 PUFA before sap lesion was able to protect the DG by the astrogliosis, as demonstrated by the significant decrease of GFAP^+^ cells in n-3 PUFA sap mice respect to the oil sap group (*p* = 0.03, Figure 5G,I).

#### 2.3.3. Hippocampal Choline Acetyltransferase (ChAT) and Vesicular Acetylcholine Transporter (VAChT) Expression

An extensive loss of ChAT immunoreactive neurons in the medial septum was verified by inspection in sap-lesioned mice (Supplementary Materials Figure S5).

As regard the densitometric analysis of VAChT immunostaining in the different hippocampal regions, the two-way ANOVA analysis (diet x lesion) revealed significant effect of both lesion (CA1: F_1,16_ = 9.808, *p* < 0.0001; CA3: F_1,16_ = 20.84, *p* < 0.001; DG: F_1,16_ = 42.23, *p* < 0.0001), diet (CA1: F_1,16_ = 31.28, *p* < 0.0001; CA3: F_1,16_ = 7.853, *p* < 0.05; DG: F_1,16_ = 22.26, *p* < 0,001), as well the interaction (CA1: F_1,16_ = 31.61, *p* < 0.0001; CA3: F_1,16_ = 7.801, *p* < 0.05; DG: F_1,16_ = 16.71, *p* < 0,001). Moreover, in the different hippocampal regions post-hoc comparisons showed a significant reduction of VAChT expression in oil sap-lesioned group compared with the other groups (CA1: oil sham vs. oil sap, *p* < 0.0001; oil sham vs. n-3 PUFA sap, *p* < 0.05; oil sap vs. n-3 PUFA sham, *p* <0.0001; CA3: oil sham vs. oil sap, *p* < 0.001; oil sap *vs* n-3 PUFA sham, *p* < 0.001; DG: oil sham *vs* oil sap, *p* < 0.0001; oil sap *vs* n-3 PUFA sham, *p* < 0.0001).

**Figure 6:**
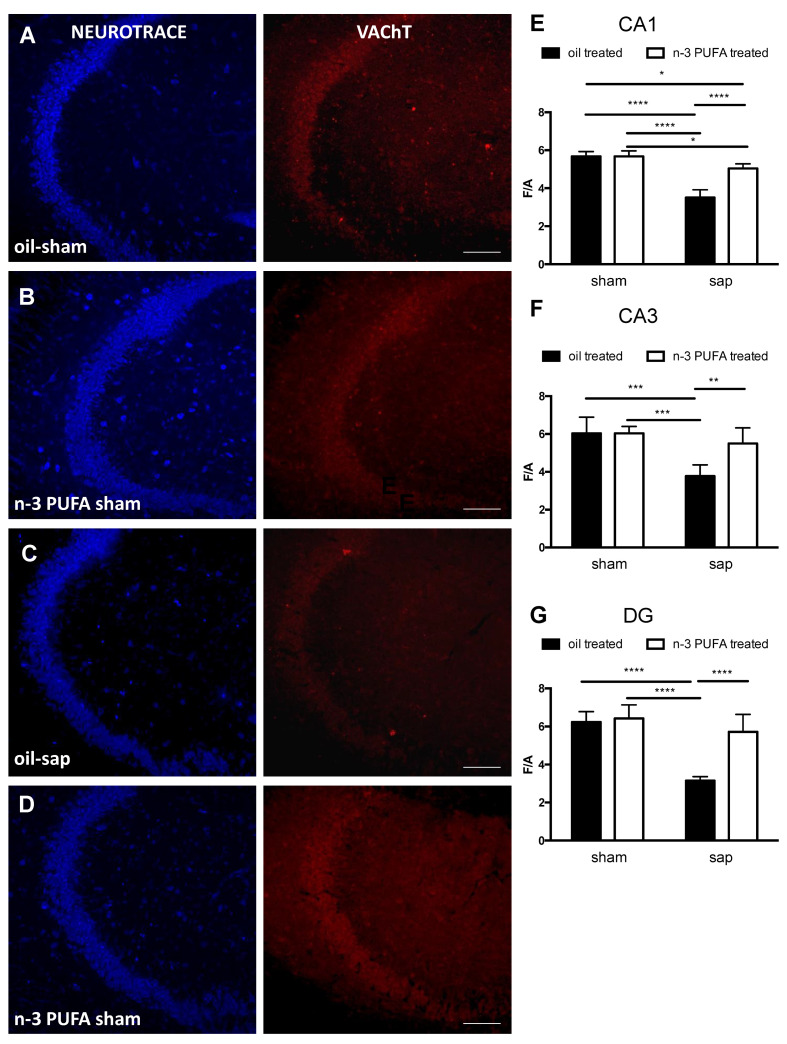
Densitometric hippocampal VAChT expression. (**A**–**D**). Confocal images from the hippocampus, namely CA3 region, of oil sham, n-3 PUFA sham, oil sap and n-3 PUFA sap mice stained with Neuro-trace (blue) and VAChT (red) showing the expression of VAChT immunostaining. (**E**–**G**). Representative densitometric graph of the expression levels of VAChT in CA1 (**E**), CA3 (**F**) and dentate gyrus (DG; G) of oil sham, n-3 PUFA sham, oil sap and n-3 PUFA sap mice. The F/A ratio defines mean fluorescence of individual samples (**F**) normalized to total Area (**A**). Data of the four experimental groups are depicted as mean and SEM (n = 5/group). * *p* < 0.05, ** *p* < 0.01, *** *p* < 0.001, **** *p* < 0.0001. Scale bar: 50 µm.

Indeed, while the VAChT expression was not significantly different in the hippocampi from sham-lesioned groups (oil sham and n-3 PUFA sham), it was significantly different in the sap-lesioned groups (CA1: oil sap vs. n-3 PUFA sap, *p* < 0.0001; CA3: oil sap vs. n-3 PUFA sap: *p* < 0.01; DG: oil-sap vs. n-3 PUFA sap, *p* <0.0001) with a VAChT expression in the n-3 PUFA sap comparable to sham groups, except for CA1 (n-3 PUFA sham vs. n-3 PUFA sap, *p* < 0.05) (Figure 6).

### 2.4. Western Blot Analysis of Hippocampal VAChT and GFAP Levels

**Figure 7:**
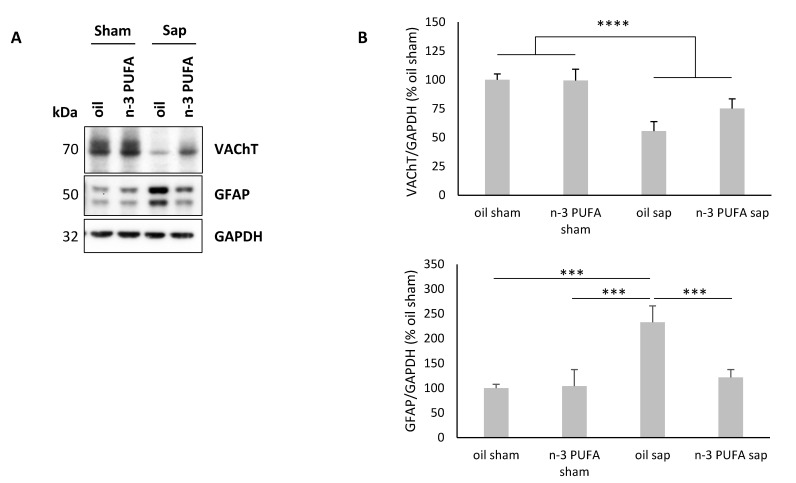
Hippocampal VAChT and GFAP immunoblotting results. (**A**) Immunoblots of total hippocampal proteins from 24-month-old mice. (**B**) The histogram shows densitometric quantification of changes in gray values, expressed as % of oil sham group. Data of the four experimental groups are depicted as mean and SEM. GAPDH was used as loading control. (VAChT analysis: oil sham, n = 11; n-3 PUFA sham, n = 10; oil sap, n = 8; n-3 PUFA sap, n = 7; GFAP analysis: oil sham, n = 11; n-3 PUFA sham, n = 9; oil sap, n = 9; n-3 PUFA sap, n = 7). Statistical significance of the post-hoc comparisons between oil sap and the remaining groups: *** *p* < 0.001; **** *p* < 0.00001.

Immunoblot analysis showed an extensive reduction of VAChT in the hippocampus of sap-lesioned mice (Figure 7), thus indicating a reduction of cholinergic terminals, in line with the abovementioned immunofluorescence densitometric results (Figure 6). Specifically, regarding the immunoblot analysis of VAChT expression in the hippocampus, the two-way ANOVA (diet x lesion) revealed a significant lesion effect (F_1,32_ = 28.353, *p* < 0.00001), while diet (F_1,32_ = 2.19, *p* = 0.15) and interaction (F_1,32_ = 2.48, *p* = 0.13) were not significant.

To examine whether the n-3 PUFA exert an anti-inflammatory activity in our mouse model, we analyzed the GFAP expression (Figure 7). The two-way ANOVA performed on GFAP levels showed significant diet and lesion effects, and significant diet x lesion interaction (diet: F_1,32_ = 7.36, *p* = 0.01; lesion: F_1,32_ = 14.43, *p* < 0.001; diet x lesion: F_1,32_ = 8.5, *p* = 0.006). Post-hoc comparisons showed that sap-lesioned mice pre-treated with n-3 PUFA showed reduced levels of GFAP, compared to oil-treated animals, whereas n-3 PUFA had no effect on sham-lesioned mice (oil sham vs. oil sap, *p* < 0.001; n-3 PUFA sham vs. oil sap, *p* < 0.001; oil sap vs. n-3 PUFA sap, *p* < 0.001).

These findings prove that n-3 PUFA pre-treatment blunts hippocampal astrogliosis in sap-lesioned mice.

## 3. Discussion

The loss of integrity of the basal forebrain cholinergic system is a consistent hallmark of AD [90,91]. By counteracting acetylcholine reduction in the synaptic cleft cholinesterase inhibitors, such as donepezil, are currently used as a symptomatic treatment to attenuate AD-related cognitive deterioration [92,93]. Anyway, pharmacological treatments of this type are not very efficient on AD progression [8]. Conversely, the promotion of lifestyle modifications, for example through “nutrigeroprotective” interventions, seems a more productive approach to prevent or delay the onset of AD symptomatology [94].

Remarkably, recent meta-analyses demonstrated that higher fish intake is associated with a slower memory decline in older people and a lower risk of dementia and AD [95,96]. Several preclinical studies confirm these beneficial effects against AD-related cognitive deterioration [2,3,38,97]. Anyway, randomized controlled clinical trials did not provide consistent evidence to support the effectiveness of n-3 PUFA supplementation in improving cognitive function in AD patients in the short and medium term [98]. Thus, in order to promote preventive interventions for elderly people at risk of dementia or in the prodromal stages, it seemed interesting to study the neuroprotective potential of n-3 PUFA when supplemented before the onset of AD pathology.

To address this issue, in the present study we investigated whether a pre-treatment with n-3 PUFA was able to prevent behavioral and/or morphological deficits induced by basal forebrain cholinergic depletion in aged mice. The immunotoxic lesion of the basal forebrain through saporin provides a valid animal model to partially mimic AD pathology by provoking a selective and permanent removal of basal forebrain cholinergic inputs to the hippocampus, the entire cortical mantle, the amygdala and the olfactory bulb [13]. Overall, here we found that n-3 PUFA pre-treatment was effective in counteracting some functional and morphological deficits induced by the cholinergic depletion. Specifically, n-3 PUFA pre-treatment restored explorative and mnesic functions, reduced hippocampal volume loss and astrogliosis, and increased neurogenesis in aged lesioned mice.

The n-3 PUFA pre-treatment did not influence any behavioral or biochemical parameter in sham-lesioned aged mice. We previously demonstrated that naïve aged mice treated with n-3 PUFA *during* behavioral testing showed enhanced memory performance and coping skills, without variations in anxiety levels [39,40]. In addition, n-3 PUFA treatment during aging increased neurogenesis, reduced astrogliosis and preserved hippocampal and prefrontal volumes [39,40]. Thus, while n-3 PUFA treatment *during* testing improves behavioral performance and hippocampal morphology in unlesioned aged animals, a similar treatment *before* testing exerts neuroprotective action in aged mice only in the presence of the cholinergic lesion.

Cholinergic depletion *per se* reduced hippocampal volume (specifically of the whole hippocampus and Ammon’s Horn, but not of DG) and increased hippocampal astrogliosis in agreement with findings in rats and AD patients. In fact, recently Dobryakova et al. [99] found that 192 IgG-saporin lesions in rats resulted in a significant CA3 neuronal loss accompanied by microglial proliferation, dense gliosis, and strong activation of astrocytes in the DG and white matter. These effects were not linked to a direct action of the immunotoxin because of the lack of the nerve growth factor receptors in the hippocampus. Therefore, the selective death of cholinergic neurons in the medial septal area and diagonal band can be considered the major factor leading to the specific vulnerability of Ammon’s Horn neurons, both in rats [99] and in mice (present data). Interestingly, some AD cases are associated with a neuronal loss in specific Ammon’s Horn sub-fields (e.g., CA3; [100,101]). Additionally, activated microglia and astrocytes [102] and high proinflammatory cytokines levels [103,104] are present in *post-mortem* AD brains, indicating the involvement of neuroinflammatory processes in the pathogenesis of the disease.

No lesion effect on neurogenesis was found in aged mice, in line with a previous study in adult mice [105]. Moreover, the lesion reduced explorative behavior and impaired retention memory, but it did not affect anxiety levels, self-care, and motivational and social behaviors, olfactory discrimination, and coping responses. The present behavioral findings are consistent with the altered locomotion [11,12,106], the memory deficits [11,15,107,108], and the lack of effects on anxiety levels [106,108,109,110] described following cholinergic depletion in adult rodents. The lack of deficits in motivational or rewarding behaviors (such as grooming in ST, eating the palatable food pellet in HFT, affiliative behaviors in SI) following the immunotoxic lesion is likely related to the sparing of striatal circuits, not involved in sap-induced degeneration [11,111,112].

Interestingly, n-3 PUFA pre-lesional supplementation prevented the decrease in explorative behavior in EPM and the retention memory deficits in POFC induced by cholinergic depletion. These behavioral improvements were accompanied and possibly sustained by the preserved morphological features of the hippocampal formation. In fact, in n-3 PUFA sap group we observed increased neurogenesis in the DG, preserved Ammon’s Horn volumes and reduced astrogliosis in comparison to oil sap group.

Beneficial effects of n-3 PUFA treatment on behavioral performance, especially on memory functions, during aging have been demonstrated in many experimental models of pathological and non-pathological aging [2,3,23,38,39,40], but this is the first study investigating the behavioral effects of pre-lesional treatment with n-3 PUFA in aged mice and also the first one evaluating astrogliosis, neurogenesis, and volume modifications in the hippocampus.

In consonance with the counteracting action of n-3 PUFA against brain atrophy previously reported in rodents [2,25,38,39,40] and humans [44,48,51,113], the n-3 PUFA supplementation prevented the decrease in the volume of the Ammon’s Horn following the cholinergic depletion. This neuroprotective effect was observed in the presence of global hippocampal atrophy and lack of volumetric modifications in the DG. Specific volumetric preservation of Ammon’s Horn in n-3 PUFA lesioned mice may be related to the already demonstrated n-3 PUFA neuroprotective properties, such as the reduction in apoptosis, neuronal density loss and glial degeneration in aged mice [23,39]. Moreover, although neurogenic proliferation is a prerogative of DG, newborn neurons may migrate into CA1 and CA3 regions of the hippocampus, where they integrate into existing circuitry and contribute to repair [114]. Therefore, in the present study the increased Ammon’s Horn volume of n-3 PUFA sap mice can be considered to be an effect of both a reduced neuronal death promoted by the reduced neuroinflammatory state of the hippocampal circuits (i.e., reduced hippocampal astrogliosis) and the potential migration of the newborn neurons from DG. This neuronal finding may constitute the structural basis of the preserved mnesic performance of n-3 PUFA sap group by virtue of the multiple implications of the hippocampal neurogenesis in mnesic retention and associative learning [115]. Thus, n-3 PUFA appear to enhance the resilience of specific hippocampal regions to maintain mnesic functions.

An important aspect of the increased n-3 PUFA-induced neurogenesis was its occurrence in the context of decreased hippocampal neurodegeneration indexes, such as reduced astrogliosis and increased VAChT levels. Since as known astrocytes undergo multiple molecular and morphological changes, astrogliosis can exert both beneficial and detrimental effects in a context-dependent manner determined by specific molecular signaling cascades, representing a ubiquitous hallmark of central nervous system pathology [116]. Moreover, aging can cause a loss of normal function in astrocytes which contributes to a heightened inflammatory state [117]. AD brains are also characterized by prominent reactive astrogliosis due to destruction of nearby neurons [118]. In this study, the hippocampal astrogliosis was detrimentally enhanced by the cholinergic depletion that elicited concomitant mnesic impairment. Notably, n-3 PUFA pre-treatment exerted a clear neuroprotective, anti-inflammatory role by lowering its increased hippocampal levels (in line with previous studies [23,39,119]), which was accompanied by a concurrent improvement of mnesic performance. In addition, the n-3 PUFA synaptogenic properties may explain the increased VAChT levels in the DG and CA3 [23,120,121]. This partial cholinergic transmission recovery can be probably due to a compensatory sprouting of cholinergic terminals.

Anyway, the mechanisms of the n-3 PUFA neurogenic action have been not yet conclusively described. We advance that the increased neurogenesis in the DG we observed could be caused by a specific interaction between the cholinergic lesion and the pre-treatment with n-3 PUFA. In several studies it has been shown that following a brain injury there is a synaptic compensatory recruitment from neural networks to maintain the functionality affected by the lesion. For example, traumatic brain injury [122,123] and cerebral ischemia [124,125] stimulate hippocampal neurogenesis to compensate for neuronal loss. Also, in transgenic AD mice as well as in AD patients, hippocampal neurogenesis is increased either in response to the impaired neurotransmission or to disease-induced neuronal loss [126,127].

Adult neurogenesis is regulated by a multitude of extracellular cues, including hormones, growth factors and neurotransmitters [128]. Interestingly, n-3 PUFA enhance cholinergic transmission and neuronal membrane fluidity, both important modulators of neurogenesis during aging [16,25,129,130]. It is well-known that there is a high cholinergic innervation in the subgranular zone of the hippocampus and that the newborn cells can respond to cholinergic inputs [131]. In fact, systemic administration of the cholinergic agonist physostigmine increases neurogenesis in the DG and the new cells express muscarinic M1 and M4 receptors [132]. Moreover, the α7-nAChR cholinergic receptors are involved in the survival and morpho-functional maturation of newborn neurons [133]. Thus, the cholinergic stimulation of neurogenesis could have been occurred both during pre-lesional n-3 PUFA pre-treatment (*neuroplastic promoting effect*), as indicated by Willis and colleagues [130], and after cholinergic lesion (*neuronal loss compensatory effect*), as indicated by the present VAChT findings. The interaction between neurogenesis and cholinergic transmission is supported by the results of another ongoing study (data not shown) in which n-3 PUFA supplementation has been administered in aged mice *after* cholinergic depletion, and histological analyses on newborn neurons carried out immediately after the end of the n-3 PUFA treatment. We have observed an n-3 PUFA potentiating effect on neurogenesis in both sham and sap-lesioned groups, thus suggesting that the neurogenic effects of the supplementation are visible during treatment and fade away when it is over.

Notably, since the neurogenesis tends to have an age-related decline [134], the aged mice used in the present study (around 24 months of age) presumably expressed extremely low levels of neurogenesis that were significantly increased only in the case of concomitance between cholinergic lesion and n-3 PUFA pre-treatment. The n-3 PUFA pre-treatment may be hence interpreted as a form of *neuroplasticity reserve* to be spent in the case of increased neurodegeneration, as it occurs in the presence of cholinergic depletion inducing hippocampal volume loss and astrocytosis increase.

## 4. Materials and Methods

### 4.1. Animals

C57BL/6 male mice (n = 42) purchased from Envigo (S. Pietro al Natisone, Italy) were used. At their arrival, the animals were 8–9 months old and they were all ex-breeders. The animals were group-housed (3–4 mice/cage) with temperature (22–23 °C) and humidity (60 ± 5%) controlled, under a 12:12 h light/dark cycle, with food and water freely available throughout the study.

Animals were randomly assigned to the following experimental groups:

- sham-lesioned aged mice pre-treated with olive oil (oil sham, n = 12);
- sham-lesioned aged mice pre-treated with n-3 PUFA (n-3 PUFA sham, n = 10);
- mu-p75-saporin-lesioned aged mice pre-treated with olive oil (oil sap, n = 10);
- mu-p75-saporin-lesioned aged mice pre-treated with n-3 PUFA (n-3 PUFA sap, n = 10).

All efforts were made to minimize animal suffering and to reduce the number of mice used, in accordance with the European Union Directive of September 22, 2010 (2010/63/EU). All experiments were approved by the Italian Ministry of Health (Legislative Decree No 682/2016).

#### 4.1.1. Dietary Manipulations

To evaluate the potential neuroprotective role of n-3 PUFA in the presence of degeneration of basal forebrain cholinergic neurons during aging, 21-month old animals were supplemented by gavage for 8 weeks (5 days per week) with a mixture of n-3 PUFA (containing 39.2% DHA, 52% EPA and 6% DPA; Meaquor 900, UGA Nutraceuticals, Monza, Italy) or olive oil (used as isocaloric control containing 14.6% saturated fatty acids, 68.3% monounsaturated fatty acids and 8.7% PUFA of which 0.6% n-3 PUFA, i.e., ALA; De Cecco, Fara San Martino, Italy) at a 350 mg/kg dosage (Figure 1). We used gavage as it is the most accurate and reliable method for administering substances into the gastro-intestinal tract over other methods of oral administration. In fact, it eliminates risks of variability in intake among individual animals (which may arise when substances are administered through delivery in food and/or water). Moreover, to reduce any eventual stress induced by the gavage manipulation, it was performed by dedicated highly trained personnel.

#### 4.1.2. Lesioning Procedure

At the end of gavage period, mice were randomly subjected to intracerebroventricular (i.c.v.) injections of mu-p75-saporin (sap; Targeting Systems, San Diego, CA, USA) or 0.9% saline (sham lesion; Figure 1). Sap is used to deplete the central cholinergic system selectively. In fact, it is a selective toxin for mouse basal forebrain cholinergic neurons obtained by conjugating the ribosome-inactivating protein saporin (from the seeds of Saponaria officinalis) to a monoclonal antibody against the mouse p75 nerve growth factor receptor (anti-murine-p75). When the conjugate is internalized, sap breaks away from the targeting agent, and inactivates the ribosomes causing protein inhibition and, ultimately, cell death. I.c.v. injections of the cytotoxin produce a dose-dependent loss of ChAT activity in the hippocampus and neocortex without affecting neighboring neurons that express glutamic acid decarboxylase, calbindin, and parvalbumin.

Mice were anesthetized with a mixture of tiletamine/zolazepam (50 mg/kg Zoletil 100 i.p.; Virbac s.r.l., Milan, Italy) and xylazine (10 mg/kg Rompun i.p.; Bayer s.p.a., Milan, Italy). In the animals to be sap-lesioned (n = 20), the immunotoxin was bilaterally injected through a 10-μL Hamilton syringe in each ventricle (total dosage: 0.6μg/mouse [11,135]), coordinates: anteroposterior (AP) = −0.6 mm (from the bregma); mediolateral (ML) = ±1 mm (from the midline); dorsoventral (DV) = −2.2 mm (from the dura). The immunotoxin was injected at a rate of 0.1 μL/min (0.3 μL per side). At the end of administration, the needle was left in situ for four minutes to allow for diffusion.

In the remaining mice used as sham control (n = 22), saline (volume of 0.3 μL per side) was bilaterally injected into each ventricle with the same injection procedure.

#### 4.1.3. Behavioral Testing

Two weeks after surgery (time required to reach a stable and permanent loss of cholinergic neurons using saporin immunotoxin [11]) the mice underwent the following tests in this order: Elevated Plus Maze, Splash Test, Social Interactions, Hidden Food Test, Predator Odor Fear Conditioning, Porsolt Test (Figure 1). The tests were performed between 10:00 a.m. and 06:00 p.m. The animals were subjected to handling habituation prior to behavioral testing.

#### 4.1.4. Elevated Plus Maze

The Elevated Plus Maze (EPM) is a validated test to measure anxiety and locomotor activity in mice, due to their natural aversion to open spaces [39,40,70]. The maze consisted of four arms of 30-cm length and 5-cm width each. Two opposing arms are enclosed by walls 15-cm high. The elevation of the structure is 60 cm. During a 5-min trial, each mouse was placed into the central platform (5 × 5 cm) of the apparatus and allowed to freely explore the maze. Animal performance was video-recorded by a ceiling-mounted camera and manually scored by an operator blind to mice experimental grouping by using EthoVision XT (Noldus, Wageningen, The Netherlands). The maze was cleaned with a solution of 10% ethanol between trials to remove olfactory clues.

The following EPM parameters were measured: (i) duration of time spent and (ii) total frequency of entries in the open and closed arms; iii) number of defecations.

#### 4.1.5. Splash Test

The Splash Test (ST) was performed as previously described [136,137,138]. Briefly, a 10% sucrose solution was sprayed on mice’s back, and the animals were individually placed in a plastic cage. Because of its viscosity, the sucrose solution dirties mouse fur and the animals initiate grooming behavior, i.e., the cleaning of their fur by licking or scratching. Grooming bouts included nose/face grooming (strokes along the snout), head washing (semicircular movements over the top of the head and behind the ears), body grooming (body fur licking) [139]. Duration and frequency of grooming were recorded during a 5-min trial and used as measures of self-care and motivational behavior [140,141]. The number of defecations was also recorded. The plastic cage was cleaned with a solution of 10% ethanol between trials to hide animal clues. Trials were frontally recorded by a video camera and manually scored by trained observers using a stopwatch. Number of defecations was also evaluated.

#### 4.1.6. Social Interactions

Each mouse (not isolated before testing) was habituated to a novel empty cage for 5 min prior to the test. During the test of Social Interaction (SI), an unfamiliar female (2-month old) of the same strain was placed in the male’s cage for 10 min. Females were housed in unisexual groups (n = 4 animals/cage) and were in the estrous phase when tested (as assessed by the analysis of the vaginal smear). Social, sexual and non-social behaviors [142,143] were recorded from a ceiling-mounted video camera, and then the duration and frequency of behaviors were manually scored using EthoVision XT (Noldus, Wageningen, The Netherlands). Social behaviors included: (i) sniffing partner’s head and snout, anogenital region, or any other part of the body; (ii) allogrooming (grooming the partner); (iii) traversing partner’s body by crawling over/under from one side to the other; (iv) social resting. Sexual behaviors included mounting and pelvic thrusts. We also considered non-social behaviors: (i) wall-rearing and rearing (standing on the hind limbs with or without the forelimbs against the walls of the cage); (ii) exploring the cage; (iii) jumping; (iv) self-grooming (the animal licks and scratches its own fur); (v) resting alone; (vi) immobility. Number of defecations was also recorded at the end of the interaction session.

#### 4.1.7. Hidden Food Test

The Hidden Food Test (HFT) relies on the animal’s natural tendency to use olfactory cues for foraging and is used to confirm ability to smell volatile odors [75].

For three consecutive days before the test, an odor familiarization to the palatable food used in the test was performed by putting two pellets of Kellogg’s Coco Pops Chocos per animal in the cages. Twenty hours before the test, mice were food-deprived by removing all chow pellets from the food hopper. The test began by placing a single mouse in a clean cage containing 3 cm deep of clean bedding. The mouse was allowed to acclimate to the experimental cage for 5 min. Then, the mouse was transferred to an empty clean cage, while the olfactory cue (a Kellogg’s Coco Pops Chocos pellet) was buried approximately 1 cm beneath the bedding surface, in a random corner of the experimental cage, and the surface smoothed out. Each trial started when the mouse was placed in the experimental cage containing the olfactory cue. By using a stopwatch, trained observers recorded for up to a maximum of 10 min the time spent to dig out the palatable food pellet and eat it. Animals that reached the cut-off not searching for the palatable food (e.g., because immobile, resting, wall-rearing or grooming most of the time) or not eating it were excluded from the analyses.

#### 4.1.8. Predator Odor Fear Conditioning

The exposure cage of Predator Odor Fear Conditioning (POFC) [78,144] consisted of a rectangular arena (43 cm × 25 cm × 20 cm) with Plexiglas white floor and transparent walls and ceiling. Intra-maze cues consisted of black stripes applied on the walls. Three-day-old bedding collected from adult rats’ home cages was used as aversive predator odor.

Before the experimental session, mice were habituated to the cage context in one 5-min session with no odor present (*habituation*). After 3 min, each mouse was placed in the zone opposite to the zone containing the predator odor stimuli and allowed to freely explore the cage for 5 min (*exposure session*). After 24 h, mice underwent a *context test session* in which they were exposed for 5 min to the same cage with no predator odor to evaluate retention memory. Mice behavior was frontally recorded with a video camera. By using a stopwatch, trained observers measured time spent in freezing (a state of immobilization except for respiration movements). A retention index was also calculated for each animal as the percentage of freezing time increase between context test session and habituation:

$$\;\frac{freezing\;duration\;during\;context\;test-freezing\;duration\;during\;habituation}{freezing\;duration\;during\;context\;test+freezing\;duration\;during\;habituation}*100$$

Number of defecations was also evaluated.

#### 4.1.9. Porsolt Test

Individual mice were gently placed in a glass cylinder (height 45 cm; diameter 25 cm) containing 20 cm water at 28 ± 2 °C. Although Porsolt Test (PT) is usually performed in two sessions, 24 h (or longer) apart, we tested animals in only one session, because our aim was to assess their coping strategies in a stressful condition [40,145]. Mice were maintained in the apparatus for 6 min. At the end of the test mice were removed from the cylinder, allowed to dry in a small cage placed under a heat source and returned to their home cages. The behavior exhibited by each animal during the test was recorded by using a frontally mounted video camera and, then, an observer blind to the treatment received by each animal manually scored the videos (EthoVision XT, Noldus, Wageningen, The Netherlands).

Duration and frequency of the following behaviors were taken into account:

- passive behaviors: immobility (total absence of movement); paddling (small movements of one of the posterior paws not producing displacement);
- active behaviors: swimming (large and horizontal movements of the paws leading to displacement of the body around the cylinder); climbing (vigorous vertical movements of the forepaws, directed against the wall of the tank, leading to displacement of the body around the cylinder).

#### 4.1.10. Morphological and Biochemical Analyses

At the end of behavioral testing the animals were deeply anesthetized, and brains were quickly removed after decapitation. Each brain has been divided into two hemispheres. The left hemisphere was post-fixed in 4% PAF for 17 h. Afterwards, hemispheres were equilibrated in 30% sucrose and cryopreserved at −80 °C to perform histological analyses of hippocampal neurogenesis, astrogliosis, volumes, anti-Choline Acetyltransferase (ChAT) and anti-Vesicular Acetylcholine Transporter (VAChT) expression. The right hippocampus was quickly dissected and stored at −80 °C to perform Western blot analysis for VAChT and astrogliosis quantification.

### 4.2. Histology

#### Morphological Analyses

(1) Immunocytochemistry for neurogenesis and astrogliosis

The hippocampus from hemispheres embedded in Tissue-Tek OCT (Sakura, Alphen aan den Rijn, The Netherlands) was cut by cryostat at −25 °C in 40 μm coronal serial free-floating sections. For immunofluorescence analysis, sections were then stained for multiple labeling by using fluorescent methods. After permeabilization with 0.3% Triton X-100 in PBS, the sections were incubated with 3% normal donkey serum in PBS for 16–18 h with the following primary antibodies: 1:200 goat polyclonal antibodies against doublecortin (DCX) (Santa Cruz Biotechnology, Inc. Cat# sc-8066), 1:300 goat polyclonal antibodies against SOX2 (Santa Cruz Biotechnology, Inc. Cat# 17320), 1:300 rabbit polyclonal antibodies against Glial Fibrillary Acidic Protein (Promega Cat# G5601), 1:150 rabbit monoclonal antibody against Ki67 (Lab Vision Cat# RM-9106-S). Secondary antibodies used to visualize the antigen were donkey anti-rabbit Cy2-conjugated (Jackson ImmunoResearch; Ki67), 1:100 donkey anti-goat Cy3-conjugated (Jackson ImmunoResearch Cat# 705-225-147; DCX, SOX2, GFAP).

Images of the immunostained sections were obtained by laser scanning confocal microscopy by using a TCS SP5 microscope (Leica Microsystem; Germany).

Analyses were performed in sequential scanning mode to rule out cross-bleeding between channels.

(2) Quantification of cell number

Quantitative analysis of hippocampal cell populations was performed by means of design-based (assumption-free, unbiased) stereology. Slices were collected using systematic random sampling. The hemisphere was coronally sliced in rostro-caudal direction, thus including the entire hippocampus. Approximately 40 coronal sections of 40 μm were obtained from each brain; about 1-in-6 series of sections (each slice thus spaced 240 μm apart from the next) were analyzed by confocal microscopy and (by unbiased stereological method) used to count the number of cells expressing the indicated markers throughout the rostro-caudal extent of the whole hippocampus. The total estimated number of cells positive for each of the indicated markers within the DG, CA1 and CA3 areas was obtained by multiplying the average number of positive cells per section by the total number of 40 μm sections comprising the entire DG, CA1 and CA3 (spaced 240 μm) [146,147,148].

(3) Volumetric measurement

Volumes of the DG, Ammon’s Horn (*Cornus Ammonis*, CA = CA1 + CA3) and whole hippocampus were estimated by quantitative light microscopy using the Cavalieri’s method [149]. In brief, rostro-caudal sections from hippocampus of each animal (taking every sixth serial section) were mounted onto glass slide and stained with 4′,6-diamidino-2-phenylindole (DAPI) for 1 min. Stained sections were viewed at low magnification using Olympus BX53 digital photomicroscope. Digital images were then captured electronically and displayed on a computer screen. For each animal, DG, Ammon’s Horn and whole hippocampus volumes were subsequently derived by multiplying the calculated mean surface area by the section thickness (40 μm) and the total actual number of sections in which the hippocampus was present.

(4) ChAT and VAChT immunofluorescence and densitometric analyses

Sections including hippocampus were selected and incubated overnight at 4 °C in PB containing 0.3% Triton X-100 with the following antibodies: goat anti- Anti-Vesicular Acetylcholine Transporter (VAChT) (1:500; ABN100 Merk-Millipore) or rabbit anti-Choline acetyltransferase (ChAT; 1:500 Abcam). After three washes in PB, sections were incubated with Alexa Fluor 555 donkey anti-goat IgG (1:200; Life Technologies) or Alexa Fluor 555 donkey anti-rabbit IgG (1:200; Life Technologies, Carlsbad, CA, USA) and Neuro-Trace^®^ 647 Fluorescent Nissl Stain (1:400; Life Technologies) for 2 h at RT. Sections were rinsed, mounted, coverslipped and then examined using a confocal laser scanning microscope (Zeiss LSM800, Jena, Germany). The confocal image acquisitions were performed using consistent settings for laser power and detector gain.

The specificity of immunohistochemical labeling of anti-VAChT or ChAT was confirmed by omission of primary antibody and use of normal serum instead (negative controls).

For comparison of fluorescent intensities, sections from the different groups were stained in the same wells and acquired under the same conditions. The brain areas of interest—CA1, CA3, and DG—were further determined by Neuro-Trace^®^ 647 Fluorescent Nissl Stain according to Franklin and Paxinos’ Atlas of Mouse Brain [150]. Quantitative analyses of the VAChT immunoreactivity in the CA1, CA3, and DG of oil sham, n-3 PUFA sham, oil sap and n-3 PUFA sap mice was performed by densitometry. All quantitative analyses were conducted blind to the animal’s experimental group. To avoid staining variability among sections and experimental groups confocal settings for image capture were maintained constant throughout the acquisition of sections from the two groups of mice. After confocal acquisition images were exported in TIFF and analyzed with ImageJ software (http://rsb.info.nih.gov/ij/; National Institutes of Health, Bethesda, MD, USA). The background signal was determined in a non-stained area. The threshold was adjusted according to the background signal and kept constant between sections. VAChT-associated signal was quantified by manually outlining the areas of interest. Mean signal intensity (F) of the marker of interest was performed on one squared frame (200 µm per side) per area of interest on 5 sections sampled to cover the hippocampus rostro-caudal extent entirely (n = 5 mice/group). The F/A ratio defines mean fluorescence of individual samples (F) normalized to total cellular surface (A) [151].

### 4.3. Western Blot Analysis

#### 4.3.1. Total Protein Extraction

Hippocampal tissues were homogenized in lysis buffer containing (in mM) 320 sucrose, 50 NaCl, 50 Tris-HCl pH 7.5, 1% Triton X-100, 1 sodium orthovanadate, 5 β-glycerophosphate, 5 NaF and protease inhibitor cocktail, incubated on ice for 30 min and centrifuged at 15,000 g for 10 min [152]. The total protein content of the supernatant was determined by the Bradford method.

#### 4.3.2. Immunoblotting Analysis

Proteins were applied to SDS-PAGE and electroblotted on a polyvinylidene difluoride membrane. Immunoblotting analysis was performed using a chemiluminescence detection kit. The relative levels of immunoreactivity were determined by densitometry using the ImageJ software.

Primary antibodies: VAChT (1:500, Synaptic Systems, #139103); GFAP (1:1000, Dako, #Z0334); GAPDH (1:3000, Calbiochem, #CB1001). Secondary antibodies: goat anti-mouse IgG (1:3,000; Bio-Rad), goat anti-rabbit IgG (1:3,000; Bio-Rad), rabbit anti-goat IgG (1:3,000; Bio-Rad).

Membranes were stripped using Re-Blot Plus Strong Solution (Millipore) for 15 min at room temperature.

#### 4.3.3. Statistical Analysis

Data were tested for normality (Shapiro–Wilk’s test) and homoscedasticity (Levene’s test). When normally distributed, data were analyzed by parametric ANOVA. Two-way ANOVA (with diet and lesion as between-animal factors), or mixed model of three-way ANOVA (with diet and lesion as between-animal factors and arm/strategy as within-animal factors), followed by Duncan’s test when appropriate, were used. Morphological data on hippocampal volume, neurogenesis, and astrogliosis were analyzed by Student’s T test. When parametric assumptions were not fully met, data transformations (square root transformation; angular transformation for percentages) or non-parametric ANOVAs (Kruskal–Wallis test) were used. Differences were considered significant at the *p* < 0.05 level (Statistica 12, Statsoft).

## 5. Conclusions

n-3 PUFA administered before a selective cholinergic depletion during aging have neuroprotective beneficial effects by improving mnesic functions and exploratory behavior and reversing hippocampal neurodegeneration. These promising data indicate that n-3 PUFA could be a good candidate to supplement the diet of elderly people in order to counteract or at least slow down the onset of AD cognitive decline and neuropathology.

## Supplementary Materials

Supplementary materials can be found at https://www.mdpi.com/1422-0067/21/5/1741/s1. Figure S1. Duration (s) and frequency of grooming observed in the four experimental groups during ST (oil sham, n = 12; n-3 PUFA sham, n = 10; oil sap, n = 10; n-3 PUFA sap, n = 10). Figure S2A. Duration (s) and frequency of social behaviors observed in the four experimental groups during SI (oil sham, n = 12; n-3 PUFA sham, n = 10; oil sap, n = 10; n-3 PUFA sap, n = 10). Figure S2B. Duration (s) and frequency of non-social behaviors observed in the four experimental groups during SI (oil sham, n = 12; n-3 PUFA sham, n = 10; oil sap, n = 10; n-3 PUFA sap, n = 10). Figure S3. Average time (s) to dig out and eat the palatable food during HFT in the four experimental groups (oil sham, n = 12; n-3 PUFA sham, n = 9; oil sap, n = 9; n-3 PUFA sap, n = 8). Figure S4. Freezing time (s) during exposure session of POFC in the four experimental groups (oil sham, n = 11; n-3 PUFA sham, n = 10; oil sap, n = 10; n-3 PUFA sap, n = 10). Figure S5. Confocal images showing the Choline Acetyltransferase (ChAT; red) immunostaining in the medial septum (MS) of saline (sham; A) and sap-lesioned mice (sap; B). Note the almost complete depletion of cholinergic neurons after saporin injection compared with saline injection (sham). Scale bar: 100 µm. Table S1. Duration (s) and frequency of single social and non-social behaviors observed in the four experimental groups during SI. Table S2. Duration (s) and frequency of single strategies displayed by the four experimental groups during PT.
