# Correction: Cutuli et al. Neuroprotective Role of Dietary Supplementation with Omega-3 Fatty Acids in the Presence of Basal Forebrain Cholinergic Neurons Degeneration in Aged Mice. *Int. J. Mol. Sci.* 2020, *21*, 1741

**DOI:** 10.3390/ijms23136916

**Published:** 2022-06-22

**Authors:** Debora Cutuli, Eugenia Landolfo, Davide Decandia, Annalisa Nobili, Maria Teresa Viscomi, Livia La Barbera, Stefano Sacchetti, Paola De Bartolo, Annacarmen Curci, Marcello D’Amelio, Stefano Farioli-Vecchioli, Laura Petrosini

**Affiliations:** 1 IRCCS Fondazione Santa Lucia, 00143 Rome, Italy; eugenia.landolfo@uniroma1.it (E.L.); davide.decandia91@gmail.com (D.D.); a.nobili@hsantalucia.it (A.N.); mt.viscomi@hsantalucia.it (M.T.V.); livia.labarbera@gmail.com (L.L.B.); p.debartolo@unimarconi.it (P.D.B.); m.damelio@hsantalucia.it (M.D.); laura.petrosini@uniroma1.it (L.P.); 2 Department of Psychology, University of Rome “Sapienza”, 00185 Rome, Italy; stefano.sacchetti@uniroma1.it (S.S.); annacarmencurci@gmail.com (A.C.); 3 Department of Medical and Surgical Sciences, University “Campus Biomedico”, 00128 Rome, Italy; 4 Department of Life Science and Public Health Section of Histology and Embryology, Università Cattolica del Sacro Cuore, 00168 Rome, Italy; 5 Department of Human Sciences, Guglielmo Marconi University, 00193 Rome, Italy; 6 Institute of Biochemistry and Cell Biology, CNR, 00015 Monterotondo, Italy; stefano.fariolivecchioli@cnr.it

The authors wish to make the following corrections to this paper [1]:

We have realized inadvertent errors and typos in the reporting of the angular transformation formula in the Results section of the paper (Section 2.1.5). The authors are sorry and wish to make the following corrections to the text:

{arcsine [square root(percentage of freezing/100)]}.

These changes do not affect the meaning of the results and have no material impact on the conclusions of our paper, since the formula used for calculations was correct.

The authors would like to apologize for any inconvenience caused to the readers by these changes.

This correction was approved by the Academic Editor. The original publication has also been updated.
