# Supplementary material for: Neuroprotective Role of Dietary Supplementation with Omega-3 Fatty Acids in the Presence of Basal Forebrain Cholinergic Neurons Degeneration in Aged Mice
Source: Int J Mol Sci. 2020 Mar 4;21(5):1741. doi: 10.3390/ijms21051741 (PMC7084583; doi:10.3390/ijms21051741)
Supplement: Supplementary file 1 [file ijms-21-01741-s001.pptx]

## Slide 1
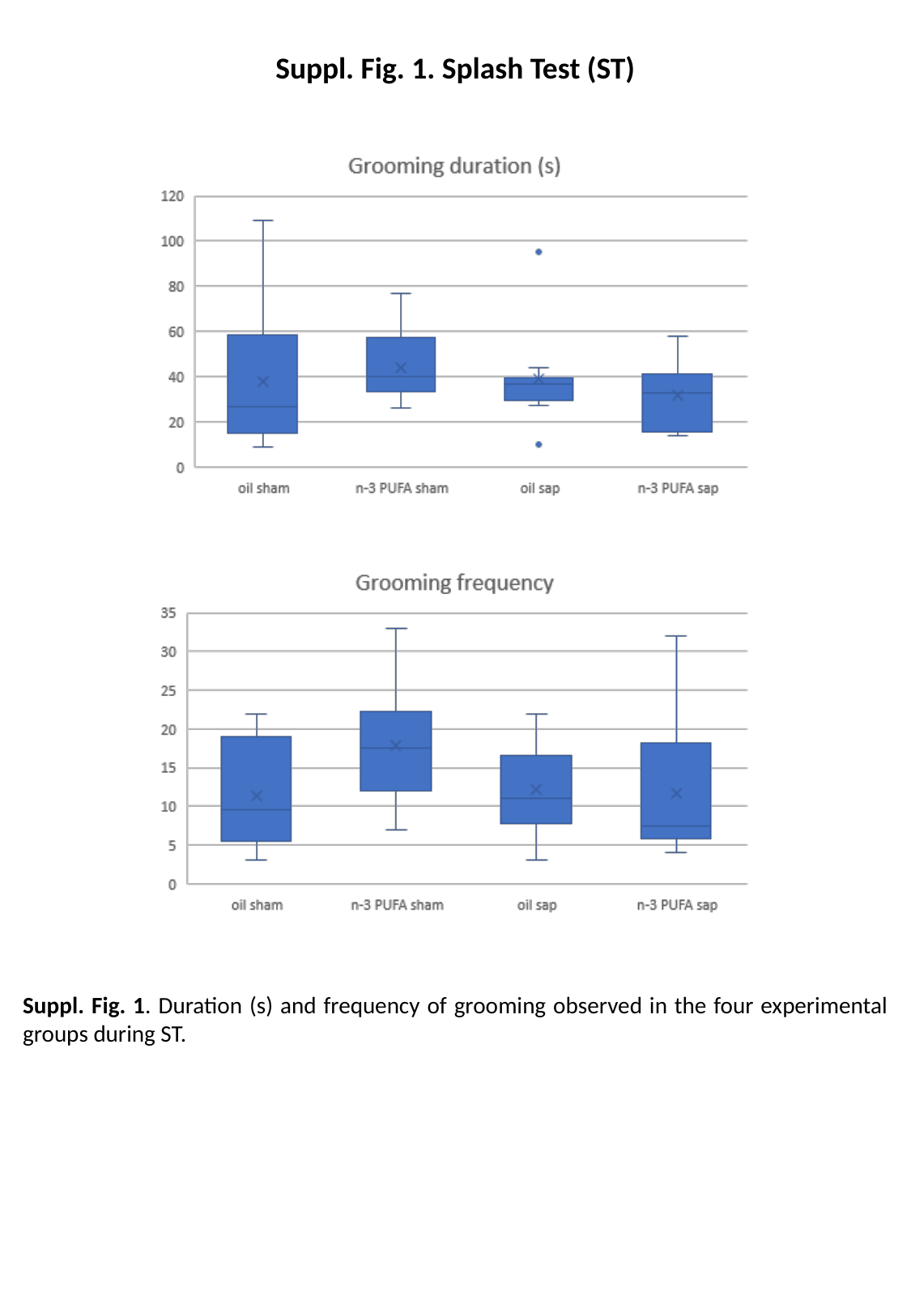

Suppl. Fig. 1. Splash Test (ST)
Suppl. Fig. 1. Duration (s) and frequency of grooming observed in the four experimental groups during ST.

## Slide 2
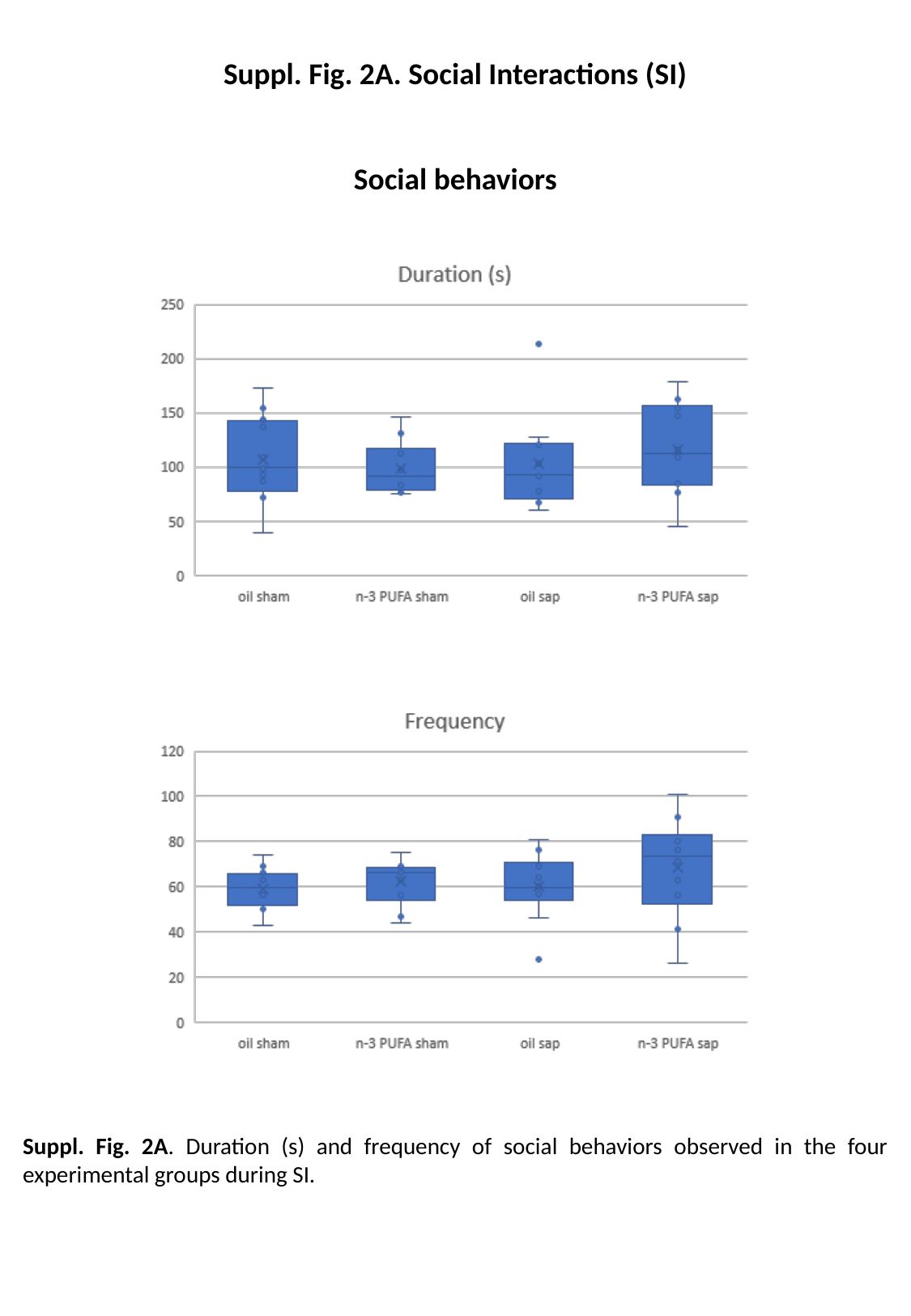

Suppl. Fig. 2A. Social Interactions (SI)
Social behaviors
Suppl. Fig. 2A. Duration (s) and frequency of social behaviors observed in the four experimental groups during SI.

## Slide 3
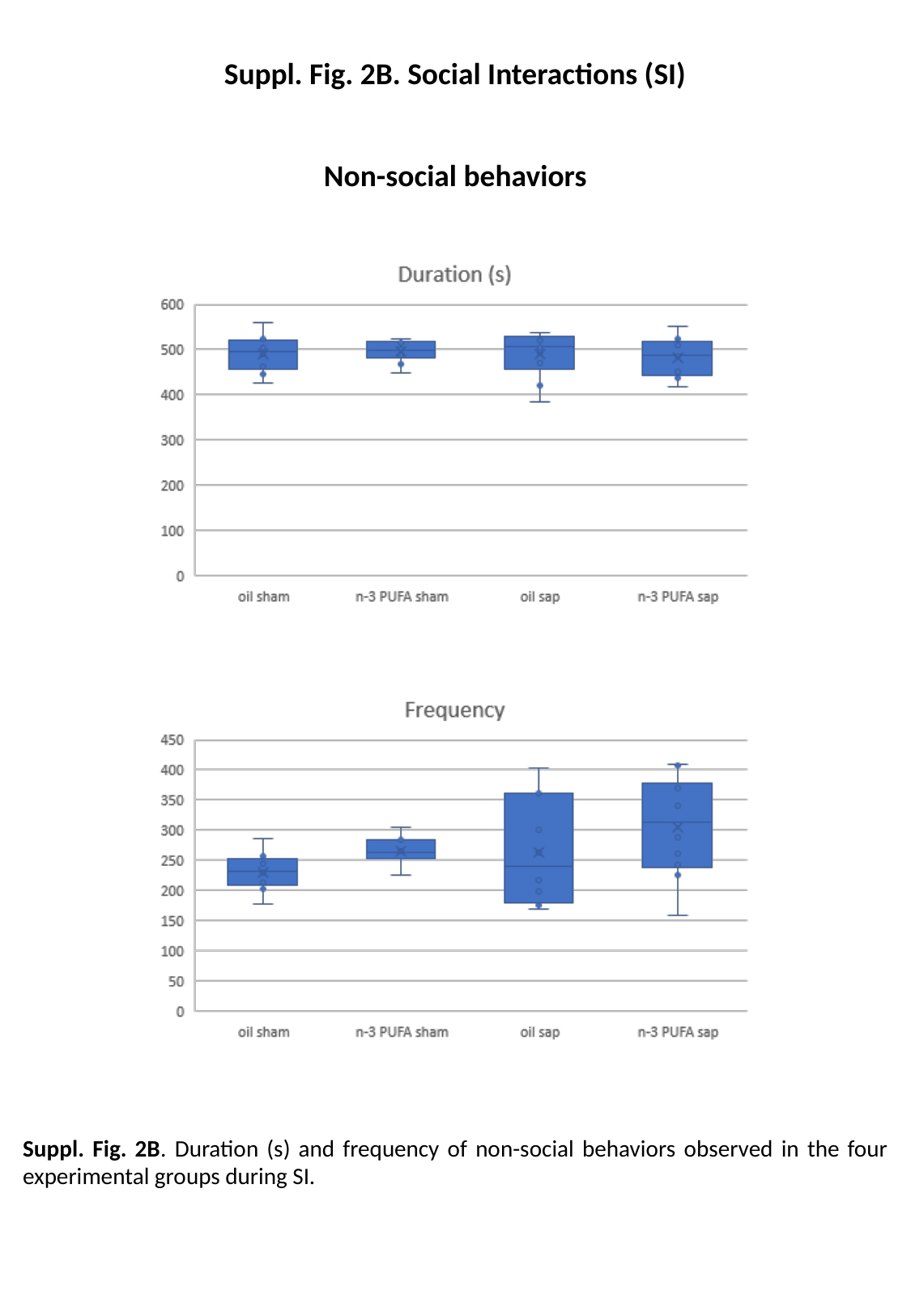

Suppl. Fig. 2B. Social Interactions (SI)
Non-social behaviors
Suppl. Fig. 2B. Duration (s) and frequency of non-social behaviors observed in the four experimental groups during SI.

## Slide 4
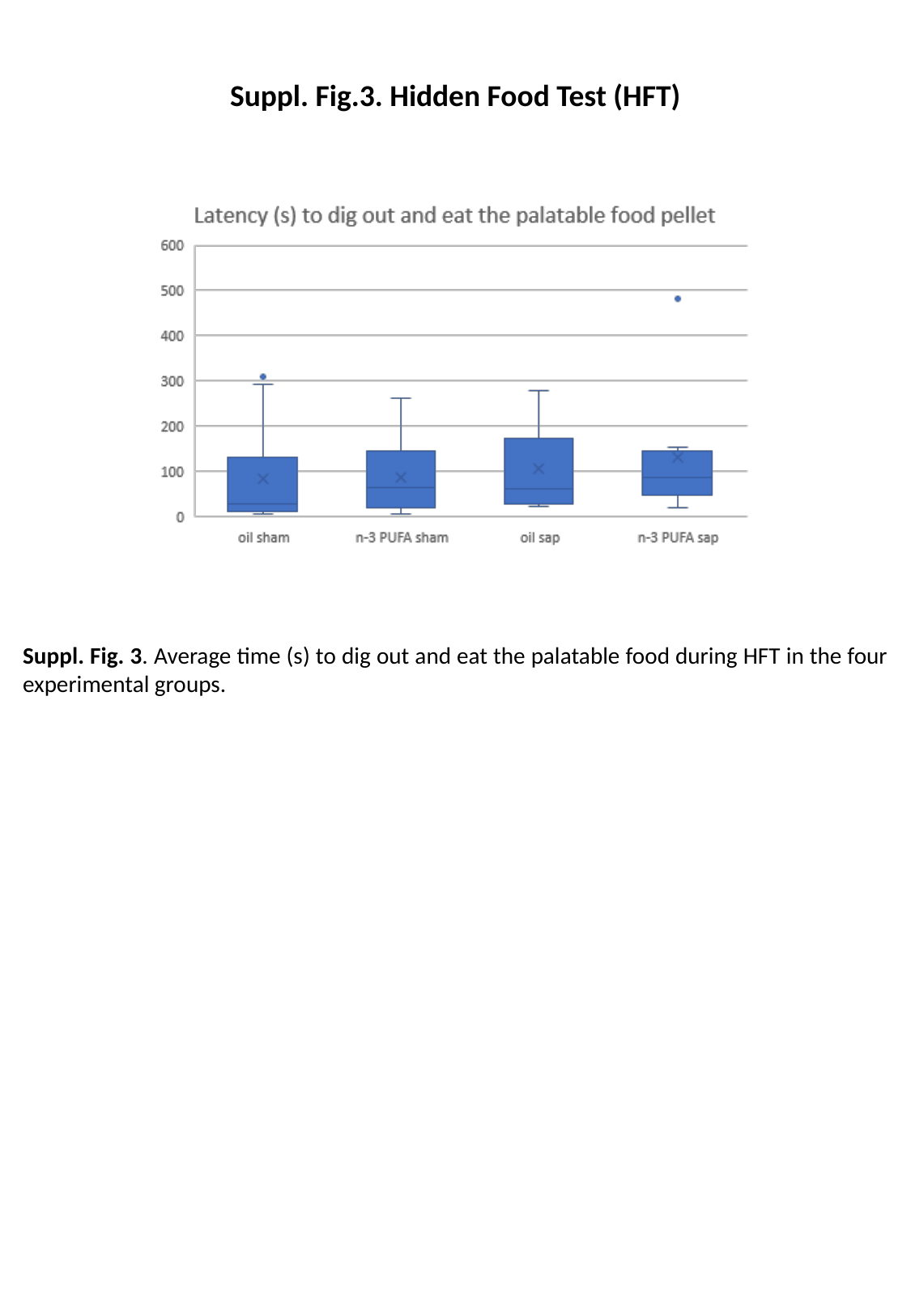

Suppl. Fig.3. Hidden Food Test (HFT)
Suppl. Fig. 3. Average time (s) to dig out and eat the palatable food during HFT in the four experimental groups.

## Slide 5
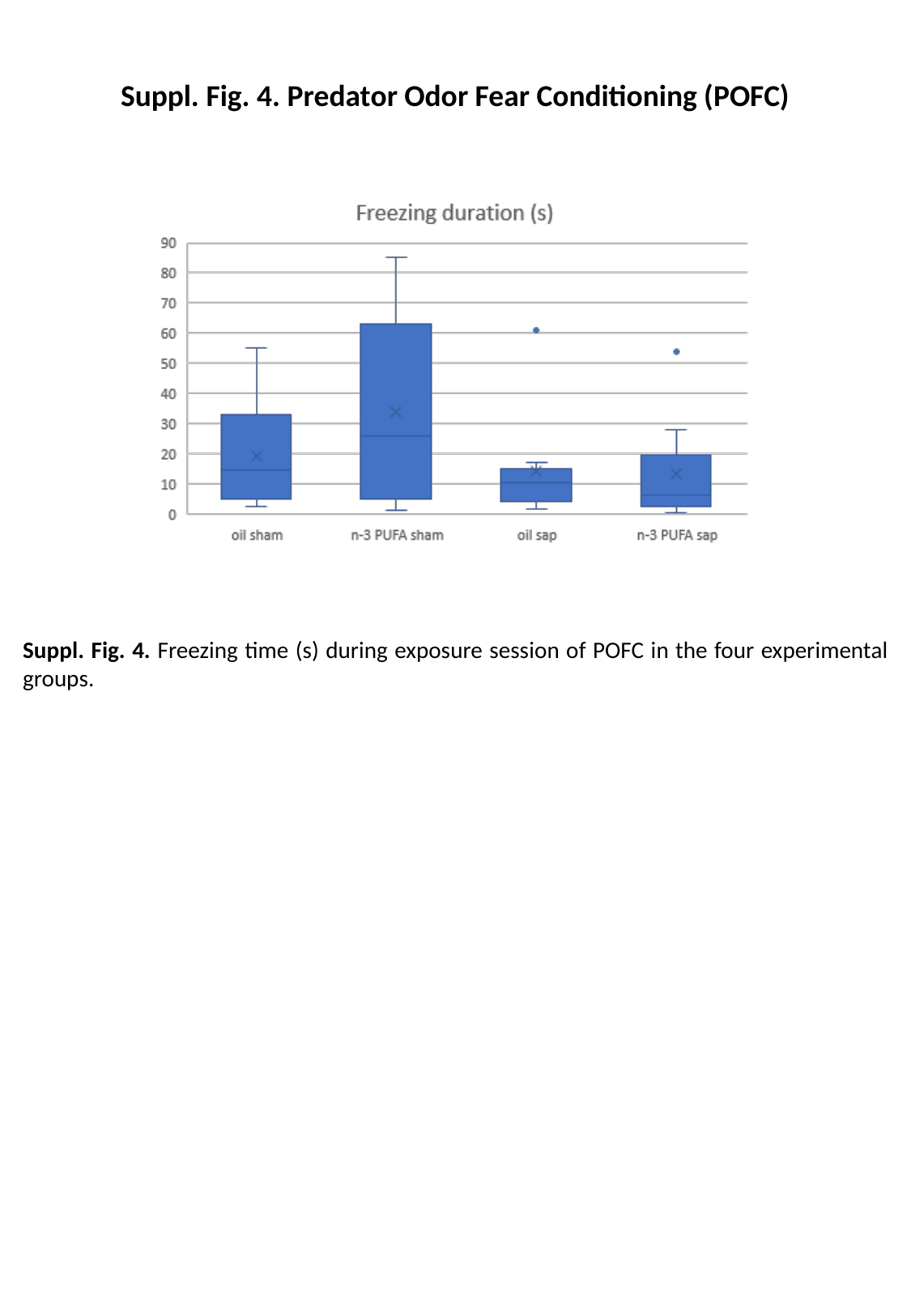

Suppl. Fig. 4. Predator Odor Fear Conditioning (POFC)
Suppl. Fig. 4. Freezing time (s) during exposure session of POFC in the four experimental groups.

## Slide 6
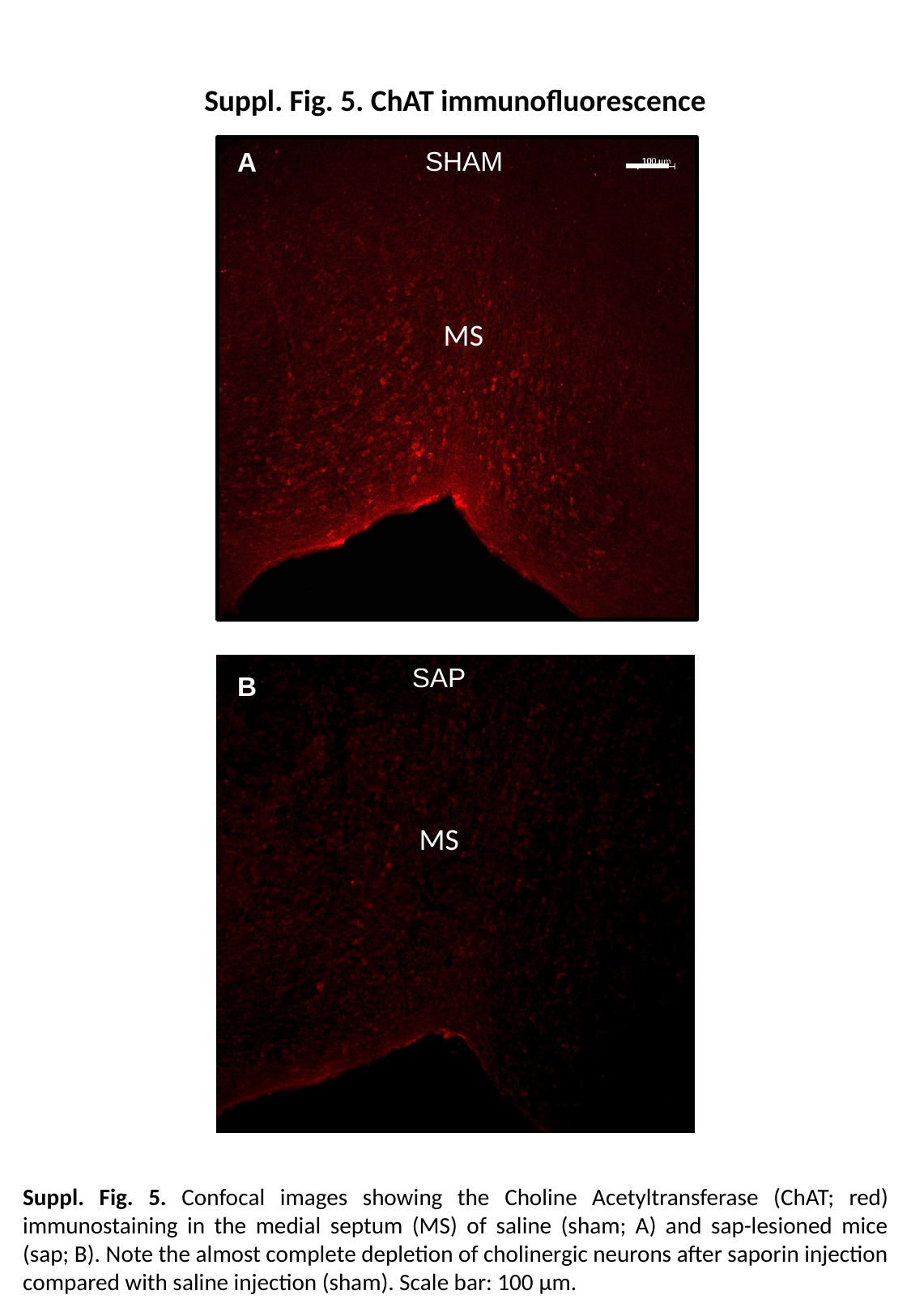

Suppl. Fig. 5. ChAT immunofluorescence
SHAM
A
MS
SAP
B
MS
Suppl. Fig. 5. Confocal images showing the Choline Acetyltransferase (ChAT; red) immunostaining in the medial septum (MS) of saline (sham; A) and sap-lesioned mice (sap; B). Note the almost complete depletion of cholinergic neurons after saporin injection compared with saline injection (sham). Scale bar: 100 µm.

## Slide 7
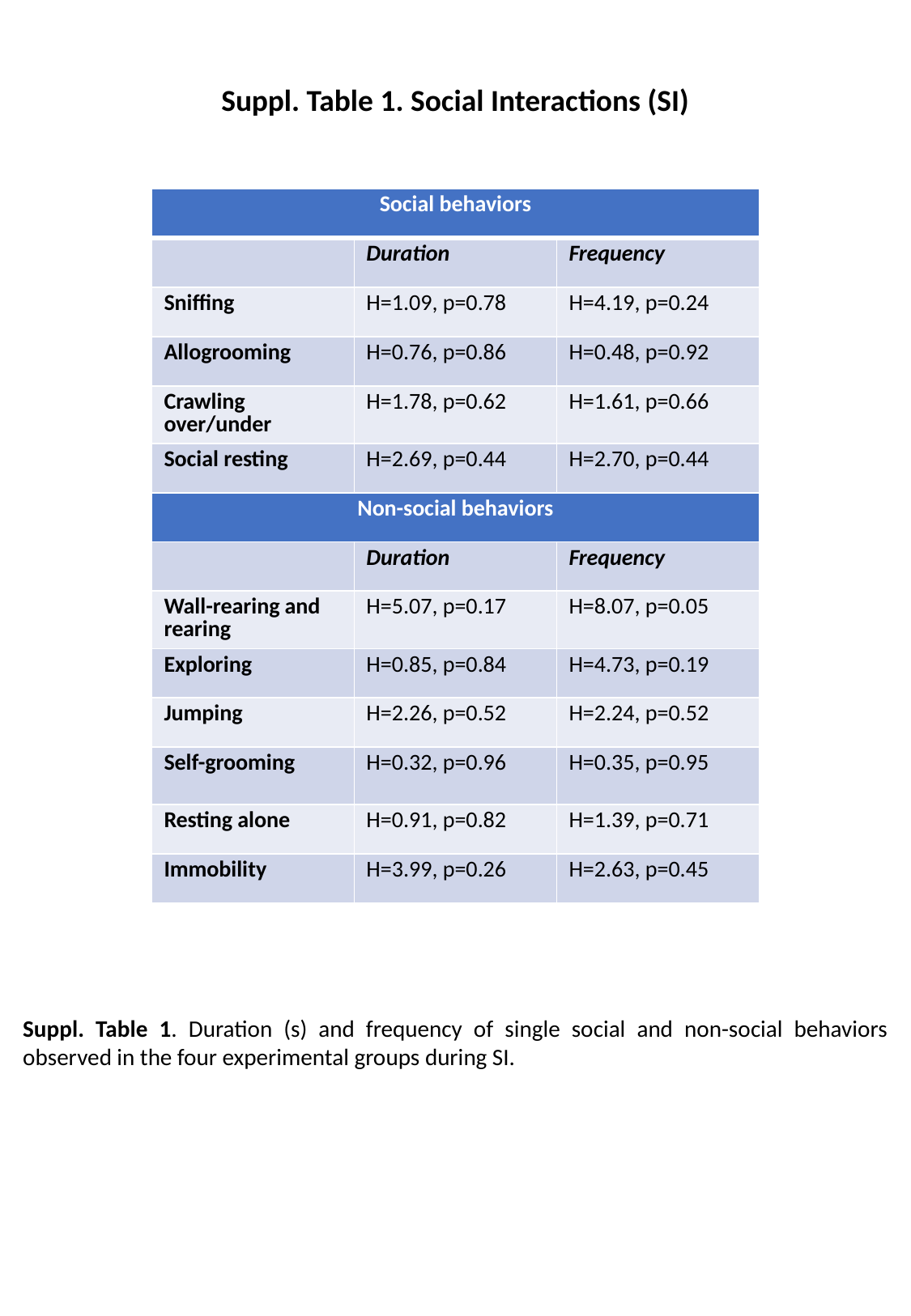

Suppl. Table 1. Social Interactions (SI)
| Social behaviors | | |
| --- | --- | --- |
| | Duration | Frequency |
| Sniffing | H=1.09, p=0.78 | H=4.19, p=0.24 |
| Allogrooming | H=0.76, p=0.86 | H=0.48, p=0.92 |
| Crawling over/under | H=1.78, p=0.62 | H=1.61, p=0.66 |
| Social resting | H=2.69, p=0.44 | H=2.70, p=0.44 |
| Non-social behaviors | | |
| | Duration | Frequency |
| Wall-rearing and rearing | H=5.07, p=0.17 | H=8.07, p=0.05 |
| Exploring | H=0.85, p=0.84 | H=4.73, p=0.19 |
| Jumping | H=2.26, p=0.52 | H=2.24, p=0.52 |
| Self-grooming | H=0.32, p=0.96 | H=0.35, p=0.95 |
| Resting alone | H=0.91, p=0.82 | H=1.39, p=0.71 |
| Immobility | H=3.99, p=0.26 | H=2.63, p=0.45 |
Suppl. Table 1. Duration (s) and frequency of single social and non-social behaviors observed in the four experimental groups during SI.

## Slide 8
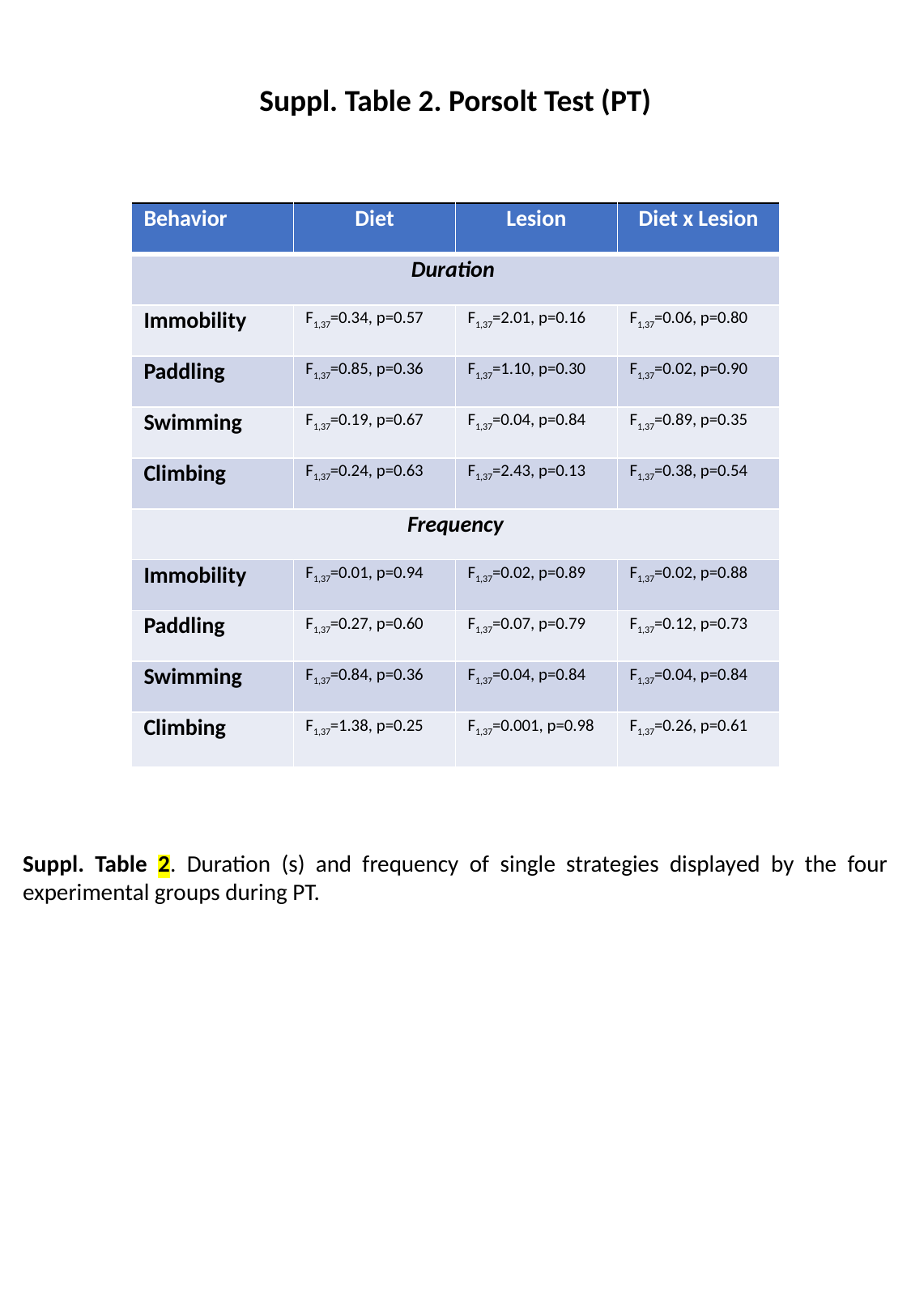

Suppl. Table 2. Porsolt Test (PT)
| Behavior | Diet | Lesion | Diet x Lesion |
| --- | --- | --- | --- |
| Duration | | | |
| Immobility | F1,37=0.34, p=0.57 | F1,37=2.01, p=0.16 | F1,37=0.06, p=0.80 |
| Paddling | F1,37=0.85, p=0.36 | F1,37=1.10, p=0.30 | F1,37=0.02, p=0.90 |
| Swimming | F1,37=0.19, p=0.67 | F1,37=0.04, p=0.84 | F1,37=0.89, p=0.35 |
| Climbing | F1,37=0.24, p=0.63 | F1,37=2.43, p=0.13 | F1,37=0.38, p=0.54 |
| Frequency | | | |
| Immobility | F1,37=0.01, p=0.94 | F1,37=0.02, p=0.89 | F1,37=0.02, p=0.88 |
| Paddling | F1,37=0.27, p=0.60 | F1,37=0.07, p=0.79 | F1,37=0.12, p=0.73 |
| Swimming | F1,37=0.84, p=0.36 | F1,37=0.04, p=0.84 | F1,37=0.04, p=0.84 |
| Climbing | F1,37=1.38, p=0.25 | F1,37=0.001, p=0.98 | F1,37=0.26, p=0.61 |
Suppl. Table 2. Duration (s) and frequency of single strategies displayed by the four experimental groups during PT.
